# A step-by-step and data-driven guide to index gender in psychiatry

**DOI:** 10.1371/journal.pone.0296880

**Published:** 2024-01-25

**Authors:** Enzo Cipriani, Eugénie Samson-Daoust, Charles-Edouard Giguère, Philippe Kerr, Consortium, Cécile Lepage, Robert-Paul Juster

**Affiliations:** 1 Centre de Recherche de l’Institut Universitaire en Santé Mentale de Montréal (CRIUSMM), Montréal, Québec, Canada; 2 Centre d’Études sur le Sexe*Genre, l’Allostasie et la Résilience (CESAR), Montréal, Québec, Canada; 3 Department of Psychiatry and Addiction, Université de Montréal, Montréal, Québec, Canada; Fondazione Policlinico Universitario Agostino Gemelli IRCCS, Universita’ Cattolica del Sacro Cuore, ITALY

## Abstract

Beyond sex as a binary or biological variable, within-sex variations related to sociocultural gender variables are of increasing interest in psychiatric research to better understand individual differences. Using a data-driven approach, we developed a composite gender score based on sociodemographic and psychosocial variables showing sex differences in a sample of psychiatric emergency patients upon admission (N = 1708; 39.4% birth-assigned females; mean age = 40 years; age standard deviation = 14). This gender score was extracted from a confirmatory factor analysis (CFI = 0.966; RMSEA = 0.044, SRMR = 0.030) and could predict a person’s birth-assigned sex with 67% accuracy. This score allowed the further identification of differences on impulsivity measures that were absent when looking solely at birth-assigned sex. Female birth-assigned sex was also associated with higher rates of mood and personality disorder diagnoses, while higher feminine gender scores were related to higher proportions of anxiety and mood disorder diagnoses. By contrast, male birth-assigned sex and higher masculine gender scores were associated with higher proportions of psychotic and substance use disorder diagnoses. Patients with undifferentiated gender scores (i.e., scoring between masculine and feminine threshold defined by terciles) were more represented in the psychotic disorder group. Considering both sex and gender in psychiatric research is essential and can be achieved even when using secondary data to index gender comprised of demographic and psychosocial variables.

## Introduction

Sex and gender are intertwined constructs that are strongly associated with health and wellbeing [1,2]. Gender is shaped in a dynamic fashion by social and cultural pressures that influence gender roles, expressions, and behaviors [3,4]. Sex and gender factors can influence physical and mental health, symptoms, and evolution presumably via stress processes and various structural pressures imposed by the social environment [5]. Despite increasing appreciation of sex and gender perspectives in health research, relatively few studies in neuroscience and psychiatry consider gender. The implementation of a tool allowing for the indirect measure of gender in available datasets could therefore be useful. The objective of the current methodological study is to describe an approach to index gendered sociodemographic and psychosocial characteristics to further understand individual differences that go beyond sex as a binary or biological variable.

### Birth-assigned sex and biological sex

Sex is often split into two concepts that are not necessarily equivalent: *birth-assigned sex* (BAS) and biological sex. BAS refers to the information that can be found on official and administrative documents, such as a birth certificate, and that was assigned at birth based on external physical characteristics (e.g., genitals). Biological sex, on the other hand, is based on physiological characteristics that are not necessarily directly observable, such as chromosomes, hormones, genetic expression, and the reproductive system [4].

Unfortunately, most research only measures BAS by offering a binary male/female choice of response. Biological sex is not binary and rather represents various continuums that can be difficult to measure. Individuals can be born with external physical characteristics that do not correspond to traditional binary sex definitions (female or male), or that can correspond to such definitions but change during puberty [6,7]. These people may be assigned a binary sex at birth due to the country or region of birth’s legislation, such as Canada or the United States of America [8]. These variations at birth, or pubertal changes in physical sexual attributes, are referred to as differences in sex development (DSD) or intersex status, with a prevalence across the world estimated to be around 1.7% [7]. Biological sex can be assessed in part through sex hormone variations (e.g., testosterone, estradiol, progesterone) as a way to represent sex continuously [9,10]. Some studies offer a third “intersex” option to their participants, although it can be limited since most people with DSD or intersex status have been assigned a binary sex at birth [8]. Some studies only ask for the sex of the participant without defining it, which can lead to confusion with the concept of gender, to which BAS may be related but still distinct for some people. This fact is present in scientific literature where sex and gender are used interchangeably [4].

### Sociocultural gender

Several operational definitions and measures of gender have emerged and evolved over time. Gender can be defined as a multidimensional construct that links gender identity, gender expression, and social and cultural expectations about status, characteristics, and behavior that are associated with sex traits [8]. Two main approaches can be distinguished in the definition and measurement of gender. One considers gender as a spectrum ranging from masculinity to femininity, while the other splits these dimensions and allows for the measurement of masculinity and femininity as separate continuums [11–14].

Furthermore, sociocultural gender is defined by the historical context in which it is measured. Indeed, some behaviors can be more or less common in a given generation rather than another. For example, social sharing of emotions depends on cultural practices and sex [15], but also fluctuates across time for a given individual regarding frequency of sharing and sharing partners [16]. These behaviors can be considered as feminine during a period and lose their gender representativity later, as they become more socially desirable in men across time [14].

Moving from the micro-level to the macro-level, gender is a multidimensional construct that encompasses different aspects that include *gender identity* (how individuals self-identify, behave, express gender, and are perceived by others), *gender relations* (the social interactions coming from the individual’s self-identification and perceived gender from others), *gender roles* (social expectations and norms associated with a given gender) and *institutionalized gender* (how power, resources and opportunities are distributed depending on gender) [17,18].

### Measuring gender in health research

In much psychiatric research, biological sex is subsumed as BAS, is often ignored, or is simply relegated as a covariate. Ideally, sex and gender should be collectively considered in study designs, analyses, and interpretations [19]. Moreover, conflating the terms “sex” and “gender” interchangeably is confusing [20,21]. It is therefore essential to disentangle sex and gender to better understand the role of biological sex and sociocultural gender in the development and manifestation of physical and mental disorders [22,23]. Furthermore, a report from the Committee on National Statistics on recommendations in measuring sex, gender and *sexual orientation* (which is orthogonal to sex and gender) underlines the fact that gender seems to be more appropriate to identify differences in lived experiences and individual characteristics than is sex [8]. Therefore, measuring gender could be more relevant to identify individual differences in mental health and wellbeing in particular.

Sex has an influence on the prevalence and symptoms of both physical and mental illnesses. For example, bipolar disorder seems to have a similar prevalence between males and females, but symptoms differ between them; males seem to experience more manic episodes while females experience more depressive episodes [24]. Such differences are partly due to hormonal differences [25], but also perhaps to sociocultural gender [20,26,27]. Sexually and gender diverse communities also experience higher risk for severe mental health disorders (e.g., psychotic episodes, major depressive disorder) due in part to greater exposure to gender-based aggression, stigma, and discrimination [21].

Literature shows that many psychiatric conditions are represented differently depending on BAS, but also depending on an individual’s gender [20,26,28–32]. Including a measure of gender in ongoing research projects could allow a better understanding of its sociocultural aspects and their role on incidence of specific conditions and/or symptomatology. Unfortunately, most research projects do not measure sociocultural gender but could greatly benefit from its inclusion to better understand within-sex variations [33]. For example, during the COVID-19 pandemic, males with low femininity reported a significant decrease in anxiety across time, meanwhile females with low femininity reported a significant increase [34]. Furthermore, among individuals with high femininity, males reported lower stress levels than females at the beginning of the pandemic [34]. In a cross-sectional study among 741 participants assessing perceived gender roles, personality traits, anxiety symptoms, depression, and suicidal thoughts/behaviors, it was identified that males and females did not differ on depressive and suicidal symptoms, but that females reported higher anxiety [35]. In the same study, individuals showing a “reversed” gender role with respect to their BAS (e.g., feminine males, masculine females) displayed more severe anxious, depressive and suicidal symptoms [35]. These few results indicate a relevant interaction between BAS and gender that could highly influence observed results, interpretation and generalizability of findings, especially in a psychiatric context.

### Composite gender indices based on psychosocial and demographic information

Advancing methods that allow for indirect measurement of gender using already available data could also help bridge the methodological gap in psychiatry. Pelletier and collaborators [36] developed a promising method to index gender with existing data. Using the GENESIS-PRAXY cohort regrouping participants with premature acute coronary syndrome, they developed a composite gender score based on an array of sociodemographic and psychosocial characteristics. Over 20 of these characteristics were selected based on presumed *a priori* sex differences within the literature. This score was created using only one measure of gender roles while the rest included sociodemographic variables such as primary earner status, responsibility for caring for children, and others. Using principal component analyses and logistic regressions (outcome male or female), they managed to create a score predicting sex with a 90% accuracy. Their composite gender score was distributed in a different fashion depending on BAS, with males’ scores clustered more along the masculine spectrum, while females’ scores clustered more along the feminine spectrum, but in a more spread-out fashion. Independent of one’s BAS, this composite gender score was correlated with acute coronary syndrome risk factors such that a greater propensity towards femininity was problematic [36].

Similarly, Smith and Koehoorn [37] also developed a gender score using secondary data from the Canadian Labour Force survey. This score was also composed of variables known in the literature as exhibiting sex differences, such as caring for children, hours of work compared to the partner, education compared to the partner, and occupational segregation (e.g., the distribution of men and women in a given occupation). This method created a score measuring gender in a sample otherwise lacking any actual gender measures. This approach allows for the utilization of gender indices in further studies with the same dataset and ultimately enables researchers to go beyond BAS as a binary variable.

### Objectives

This project has one main objective: offer a proof of concept of the possibility to create a gender score using already available datasets. It aims to bring novel methods to include in psychology, psychiatry, and neuroscience research projects to deepen our understanding of the role of sex and sociocultural gender on the brain and behavior. By including a measure of sociocultural gender, we hope to offer a much-needed holistic approach to the complex interactions observed in these disciplines.

This study aims to use a data-driven method similar to Pelletier *et al*.’s as well as Smith and Koehoorn’s method to create a composite gender score suited for psychiatric research. Our methods differ on the ground that we do not include the same variables in our procedure, and we select the variables included in our factor analyses based on preliminary statistical analyses. Nonetheless, there is some overlap regarding included variables.

We will develop this score using the Signature Biobank composed of sociodemographic, life experiences, and psychological symptoms data from 2,061 patients visiting the emergency of the largest psychiatric hospital in Quebec, Canada [38]. We will then test the composite gender score by evaluating its correlates with clinical symptoms and compare them with those identified by BAS and psychiatric symptoms that tend to differ more between men and women.

### Hypotheses

H1) A gender index will be more strongly associated with psychiatric symptoms than BAS. In this manner, patterns in masculine or feminine propensities will be different from those obtained between BAS and psychiatric symptoms.

H2) A gender index will correspond with “sex-specific” psychiatric diagnoses similarly to BAS. For example, birth-assigned female status will be associated with mood disorders and personality disorders while birth-assigned male status will be associated with psychosis and substance abuse. We likewise expect that a greater propensity towards either femininity or masculinity will be associated with these diagnostic patterns over and above BAS.

## Materials and methods

This project was approved by the research ethics board of the CIUSSS de l’Est-de-l’Île-de-Montréal. Data was obtained after ethical approval on July 25^th^, 2022. The dataset used in this project was anonymized and therefore did not allow identification of the participants, authors did not have access to such information.

### Study design and procedure

The current analysis is derived from a sample of 2,061 patients from the Signature Biobank. The Signature Biobank was developed by the Research Center of the Montreal Mental Health University Institute and consists of administrative information, mental health questionnaires, and the collection and preservation of biological samples. This information was collected from 2012 to 2020 among patients visiting the emergency services of the largest psychiatric hospital in the Canadian province of Quebec. Information from four time points were collected: during the arrival at the emergency service, at the end of the hospitalization, two months, and 12 months after hospitalization.

Additional times of measure were added to reflect the evolution of the patient’s state and offer deeper insights for research projects interested in the prediction of treatment response, for example. These longitudinal timepoints were not considered for the purpose of this study as we aimed to reach the largest sample size and are presented for informative purposes. For further information regarding these longitudinal measures, please visit the website of the Signature Biobank (https://www.banquesignature.ca/en/). Furthermore, an article presenting in deeper detail the protocol used in this biobank is currently under revision [39].

Administrative information consisted of sociodemographic data (e.g., age, BAS, socioeconomic status), current psychiatric diagnosis, and treatment information. Psychological variables measured various dimensions, such as past abuse, depressive symptoms, anxious symptoms, or psychotic symptoms using validated scales. Biological samples consisted of blood draws, hair samples, and saliva samples. The current analysis will focus on administrative and psychological data sampled during the emergency visit to develop the composite gender score and evaluate its utility in detecting individual differences in psychiatric symptom profiles.

The creation of a composite gender index involved the reduction of the number of variables included in the analyses and its fine tuning. Variables that could be considered as dependent variables in our project (e.g., psychiatric symptoms, psychiatric diagnoses) were removed from the gender score creation to avoid multicollinearity. All other administrative variables were included in preliminary analyses to test their relevance in the construction of the gender score.

#### Participants

Our sample was originally composed of 2,061 participants, out of which 39.4% were birth-assigned females, with a mean age of 40.31 years (range: 17–81; SD = 14.01). Our initial measure of sex was formulated to ask for the BAS of the person. Among these participants, 799 were diagnosed with a psychotic disorder (38.8%), 635 with a mood disorder (30.8%), 223 with a personality disorder (10.8%), 197 with an anxious disorder (including OCD and PTSD: 9.6%), 167 with a substance use disorder (8.1%), and 40 with another diagnosis (i.e., suicide attempt, dementia, eating disorder, etc.: 1.9%). Patients’ primary diagnosis was determined using the International Classification of Diseases (ICD)-10 criteria. Participants were mainly from the neighborhood of the hospital, which is considered disadvantaged. Most of the included patients reported to be White (77.5%), 240 reported to be Black (11.6%), 64 to be North African (3.1%), 51 to be Latin-American (2.5%), 16 to be South-East Asian (0.8%), 11 to be Native American (0.5%), and 73 to have other origins (e.g., Sub-Saharan, mixed race, South-Asian, etc.: 3.5%). Regarding language, 854 (41.4%) participants reported to be able to have a conversation in French only, 30 (1.5%) in English only, 1169 in both languages (56.7%), and 8 did not respond to the question.

#### Data collection

As presented in the previous section, this sample is extracted from the Signature Biobank. Before allowing the participation in the Signature Biobank, the ability to provide informed consent was established by a psychiatrist, a research nurse, and the attending clinical staff. If the participant was deemed able to provide informed consent, they were approached by a research nurse. If deemed inapt to provide consent (e.g., acute crisis), they were approached again at a more suitable time.

Participants were recruited by research nurses when entering the psychiatry emergency services. When a research nurse approached a potential participant, they systematically informed them that participating (or not) would not affect the received quality of care, nor the time they would spend in hospital. Approached prospects were kept in records by research nurses to avoid over-solicitation of patients.

Since 2012, a total of 3,411 patients were approached to participate in the Signature Biobank. Among those, one hundred and five (n = 105) were deemed inapt to consent. A total of 1,104 (32%) patients approached refused to participate in the Signature Biobank. Four hundred and forty-two (40%) of those 1,104 refused without providing specific reasons, 29% (n = 320) were not interested in participating, and 9.6% (n = 106) reported they needed more time to decide but left the hospital before providing consent. As such, a total of 2,208 patients (65%) signed a consent form. Of those, 2,107 (95%) responded to questionnaires. Some patients withdrew consent after being discharged from the hospital (n = 23, 0.1%). Of all the participants that filled the questionnaires, 2,061 responded to those used in this project. Questionnaires used were self-reported and participants responded to them directly on an iPad.

#### Measures

Presented variables in this section reflect those showing significant results in our preliminary analyses to 1) reduce the size of the article and 2) reflect what was used to build our gender score. As our goal is to present a method using already available data, our included variables do not necessarily imply an influence on gender. Nonetheless, variables included in the Signature Biobank were specifically chosen for their relationship with psychiatric symptoms.

As evoked earlier, this dataset is extracted from the Signature Biobank. The complete available variables are therefore limited by the initial protocol of the Signature Biobank that rationally proceeded to a choice of relevant variables for psychiatry research [39].

**Elaboration of the composite gender score. Sociodemographic status** (e.g., type of housing, professional status). These questions were extracted from the Canadian Community Health Survey (2006 and 2011 versions, [40]), but also from the 2006 and 2011 Canadian National Censuses.

**Birth-assigned sex** was measured as female = 0 and male = 1.

**Sleep quality and effectiveness** were measured using the Sleep Health Questionnaire [41]. This questionnaire is composed of 5 questions where participants report their sleep length and quality. It has been validated among non-hospitalized schizophrenic patients [42].

**Childhood and adolescent experiences of violence** were measured using the Childhood Experiences of Violence Questionnaire, short version, [43,44]. This questionnaire is composed of 7 items measuring emotional, physical, and sexual violence, along with experiences of emotional and physical neglect during childhood.

**Expressed aggressive behaviors** during life were measured using the Brown-Goodwin History of Aggression [45]. This questionnaire is composed of 11 items measuring aggressive behavior during childhood, teenage years, and adulthood. This questionnaire shows good psychometric properties and has been validated using the same cohort as the present study [46].

**Tobacco use** was measured using questions from the Canadian Census Health Survey and is composed of questions about global tobacco use and related behavior (e.g., number of cigarettes per day, e-cigarette use).

**Symptoms severity and mental health. Anxious symptoms** were measured using the short 6-item version of the State-Trait Anxiety Inventory (STAI-6; [47–49]). Items are composed of a 4-points Likert scale mainly oriented towards a measure of anxious state that shows excellent internal consistency.

**Depressive symptoms** were measured using the 9 items Patient Health Questionnaire (PHQ-9; [50]) that asks about the frequency and severity of depressive symptoms experienced in the past 2 weeks.

**Impulsivity** was measured using the Urgency-Premeditation-Perseverance-Sensation Seeking- Positive Urgency (UPPS-P) scale, short version [51–53]. This questionnaire is composed of 5 subscales measuring positive and negative urgency, lack of premeditation and perseverance, and sensation seeking. These subscales reflect the multidimensional conception of impulsivity.

**Psychotic symptoms** were measured using the Psychosis Screening Questionnaire (PSQ; [54]). This questionnaire is composed of 12 items measuring hypomania, intrusive thoughts, paranoia, bizarre experiences, and hallucinations.

**Alcohol use and abuse** were measured using the 10-item Alcohol Use Disorder Identification Test (AUDIT-10; [55]).

**Drug use and abuse** were measured using the Drug Abuse Screening Test (DAST-10; [56]). This questionnaire is composed of 10 items with two choices of response (yes/no), and showed good psychometric properties in the currently used sample [57].

**Social/global functioning and deficit** were measured using the World Health Organization Disability Assessment Schedule, short form, [58]. This questionnaire is composed of 12 items measuring different aspects of experienced difficulties in everyday life, such as cognition, mobility, hygiene, social life, carrying out daily tasks, and participation to daily activities. This questionnaire was validated among our psychiatric patient sample [59].

**Psychiatric diagnosis** was assessed by the referring psychiatrist and is presented following the ICD-10 criteria [60].

### Data analysis

#### Variables pre-selection procedure

To select the most promising variables to elaborate our gender index, we proceeded with several preliminary analyses. These analyses allowed us to identify polarizing variables among birth-assigned males and females which were then used to create our index. T-tests and chi-square (χ^2^) tests were performed using participant’s BAS as an independent variable. Analyses were conducted using IBM’s Statistical Package for the Social Sciences (SPSS) version 26 for Windows.

Variables showing significant differences between males and females were then selected according to three main aspects: variables with low missing data (<5%), variables showing the most significant differences, and variables not measuring psychiatric symptoms that were our main dependent variables. This process also allowed us to identify redundant variables. We aimed to satisfy two considerations: 1) to have enough variables included in the subsequent exploratory factor analysis to allow for the emergence of a stable model, and 2) to keep the most relevant variables to our research domain as dependant variables. This procedure resulted in 26 variables included in the next steps (presented in the results section).

To confirm the validity of our developed model, we separated our sample by randomly assigning 33% of the sample in a first group and 66% in a second group. The first group was used to develop our model using exploratory factor analysis (EFA) and the second group to confirm this model with confirmatory factor analysis (CFA). The final index was produced using a final CFA with the whole sample once the developed model was deemed acceptable. Some of these variables are dummy coded variables extracted from multiple choice questions (e.g., What’s your marital status? Married, divorced, widowed, separated, never married). EFA and CFA analysis were performed using R-studio 1.3.1, the lavaan package 0.6–9 [61], and psych package 2.1.9 [62].

#### Operational definition of the strategy used to develop the composite gender index

In this analysis, we used a data-driven approach rather than a literature-based approach, as was done by Pelletier *et al*. [36]. Several considerations informed this method. First, the Signature database does not contain as many variables related to more commonly identified sociocultural gender factors as the GENESIS-PRAXY cohort study investigated in Pelletier *et al*.’s original work. Mostly, missing variables reflect household and childcare dimensions, as well as some financial and job-related characteristics (e.g., primary earner of the household status, number of hours of work per week, level of responsibility for caring for children, number of hours per week spent doing housework, etc.). Also, this cohort included a known measure of gender roles, the Bem Sex-Role Inventory [11], which is not available in our dataset.

Second, if significant differences were observed between variables based on BAS, part of this variance could be due to latent effects of gender. Through their work among over 13,000 participants, Carothers and Reis [63] identified that variables that show significant differences between males and females–presented as taxonomic, or dichotomous differences–could also be understood in a dimensional approach (i.e., composed of various dimensions related to one another but also essentially distinct) indicating a gendered dimensional structure underlying observed dichotomic differences. This dimensional underlying of sex differences is what we attempt to help emerge by selecting them through preliminary statistical analyses. As such, our methodology allows for the emergence of this variability among the created gender index scores. This data-driven approach facilitates the variable selection process to compose the model and allows for the emergence of a gender score without using pre-determined gender-related variables. Furthermore, some of our variables are known to be gendered such as tobacco use [64] or educational attainment [65], for example.

At the onset, it is important to note that our data-driven approach requires careful attention to obtain a stable model and meaningful results. Therefore, we selected only the variables offering the most significant differences between males and females and removed variables that were too highly inter-correlated (*r*>0.75). We then used a back-and-forth method to define the most stable model. To do so, we included several variables in our EFA and gradually refined our included measures to obtain a stable model. Once obtained, we tested its factorial structure using CFA in our second sample.

Next, to create our composite gender index score for each individual, we used logistic regressions to determine how much our obtained factors could predict BAS. The accuracy of the probability of being female (0) or male (1) was evaluated using a receiver operating characteristic (ROC) curve and, more precisely, the area under the curve (AUC) of that curve, which measures how good the prediction is. The ROC curve evaluates the balance between sensitivity and specificity by comparing the probability to the actual sex. The AUC of the ROC curve should be above the random threshold of 0.5, where we would randomly assign the participants in each sex and approach 1, which would be a perfect score.

**Fig 1:**
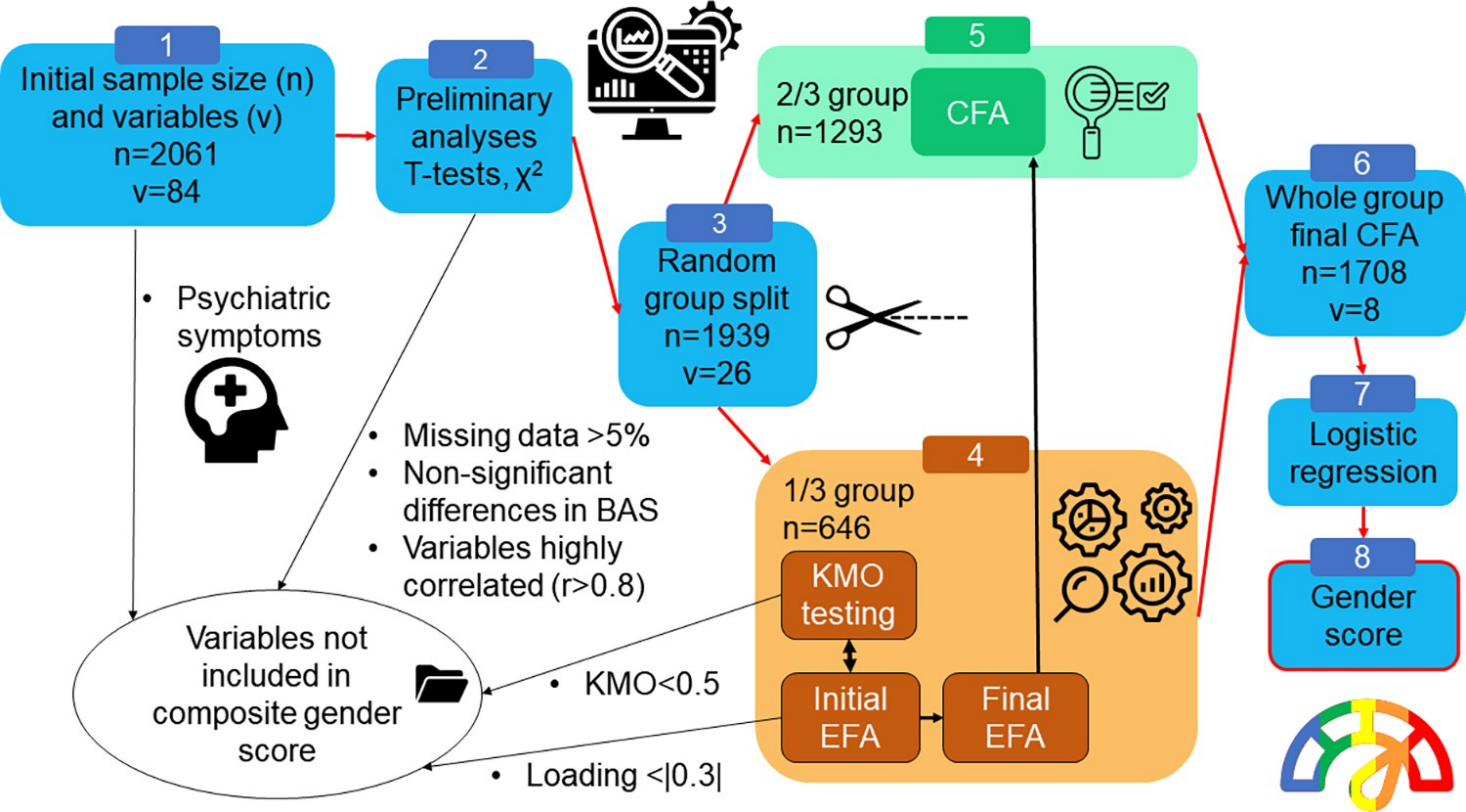
Procedure followed to produce the composite gender score. This figure presents the different steps used to create our gender score. 1) Variables measuring psychiatric symptoms were removed to be used in further analyses. 2) Variables that either contained 5% or more missing values, did not show significant differences between sexes, or were highly correlated to another variable were removed. 3) Whole sample is divided in two groups, 1/3 used in exploratory factor analysis (EFA) and 2/3 for confirmatory analysis (CFA). 4) Variables showing least adequacy for factor analysis (i.e., variables with KMO<0.5), or low factor loading (i.e., loading within -0.3 to 0.3 range), were removed. 5) Once the EFA resulted in a stable model, we proceeded with a CFA with the 2/3 of the sample to confirm EFA structure. 6) When step 5 resulted in a stable structure, a CFA with the whole sample was done. 7) We extracted each participant’s factorial scores and used them to predict BAS with logistic regressions. 8) The predicted score from the logistic regression is used as our gender score.

Several quality checks were used over this procedure and the non-attainment of one meant going back to try a new version of the model until reaching an acceptable result. These quality checks refer to two processes. First, the systematic verification of the Kaiser-Meyer Olkin (KMO) measure referring to the sample adequacy to perform factor analysis (also referred to as a Measure of Sampling Adequacy–MSA) was applied. Second, once adequate KMO was reached (i.e., KMO≥0.5), we checked for the different model fit indices such as root mean square error of approximation (RMSEA), comparative fit index (CFI), χ^2^ test of model fit and Standardized Root Mean Square Residual (SRMR) to reach acceptable thresholds (non significant χ^2^ test or a ratio χ^2^/degrees of freedom<2; CFI≥0.95; RMSEA<0.08; SRMR<0.08; [66]). As evoked earlier, the Fig 1 summarizes the procedure we followed.

#### Exploratory factor analysis

In total, 26 variables were selected to go further into the EFA procedure. These variables included sociodemographic aspects–marital status, diplomas, income sources, with whom the participants live, the status for the best job that the participant had (part-time or full time), type of housing and employment status–, sleep satisfaction and efficacy, history of aggression and childhood experience of violence. Before performing the EFA, assumptions were verified looking at KMO, sphericity, and normal distribution of our variables.

The variables included in the model were adjusted with a goal of reaching acceptable KMO assumption. Variables showing the lowest MSA were removed individually and KMO test was performed again. Acceptable threshold for KMO has been retained at 0.5 [67], although we aimed to reach 0.6 and higher. The number of factors to be tested in the EFA were determined using parallel analysis. The EFA was performed using principal axis extraction method with varimax rotation. The final model was selected when KMO reached 0.5 and most of the included variables loaded at more than |0.3| on retained factors determined by parallel analysis. From our original 26 variables, eight variables were included in the final EFA and retained for the final model, which contained four factors.

#### Confirmatory factor analysis

A CFA was conducted on the second subsample to confirm the validity of the created model with the EFA. Before the analysis, multivariate normality was tested using Mardia’s test [68]. This test evaluates the multivariate skewness and kurtosis to assess distribution normality. Model fit was determined by a χ^2^ test as well as thresholds for CFI, RMSEA and SRMR using standard cut-offs (CFI>0.95; RMSEA<0.08; SRMR<0.05; [66]). The predictive quality of this model also guided our selection using logistic regression and AUC.

#### Logistic regression

For the final model, a logistic regression was used to predict BAS using the four factors extracted during the previous analyses as independent variables. A logistic model allows predicting membership of a dichotomous variable, like BAS, with an estimated probability of being in each sex. This is a generalized linear model using the binomial family and the logit as the link function. In this study, we were not trying to construct a strong model to discriminate BAS, but rather to build a score that captures the sociocultural difference between birth-assigned males and females, so we were not expecting an AUC of the ROC curve close to 1, but at least significantly over the random threshold of 0.5. Furthermore, a prediction of BAS with 100% accuracy would not reflect a measure of sociocultural gender and therefore not bring any added value.

**Fig 2:**
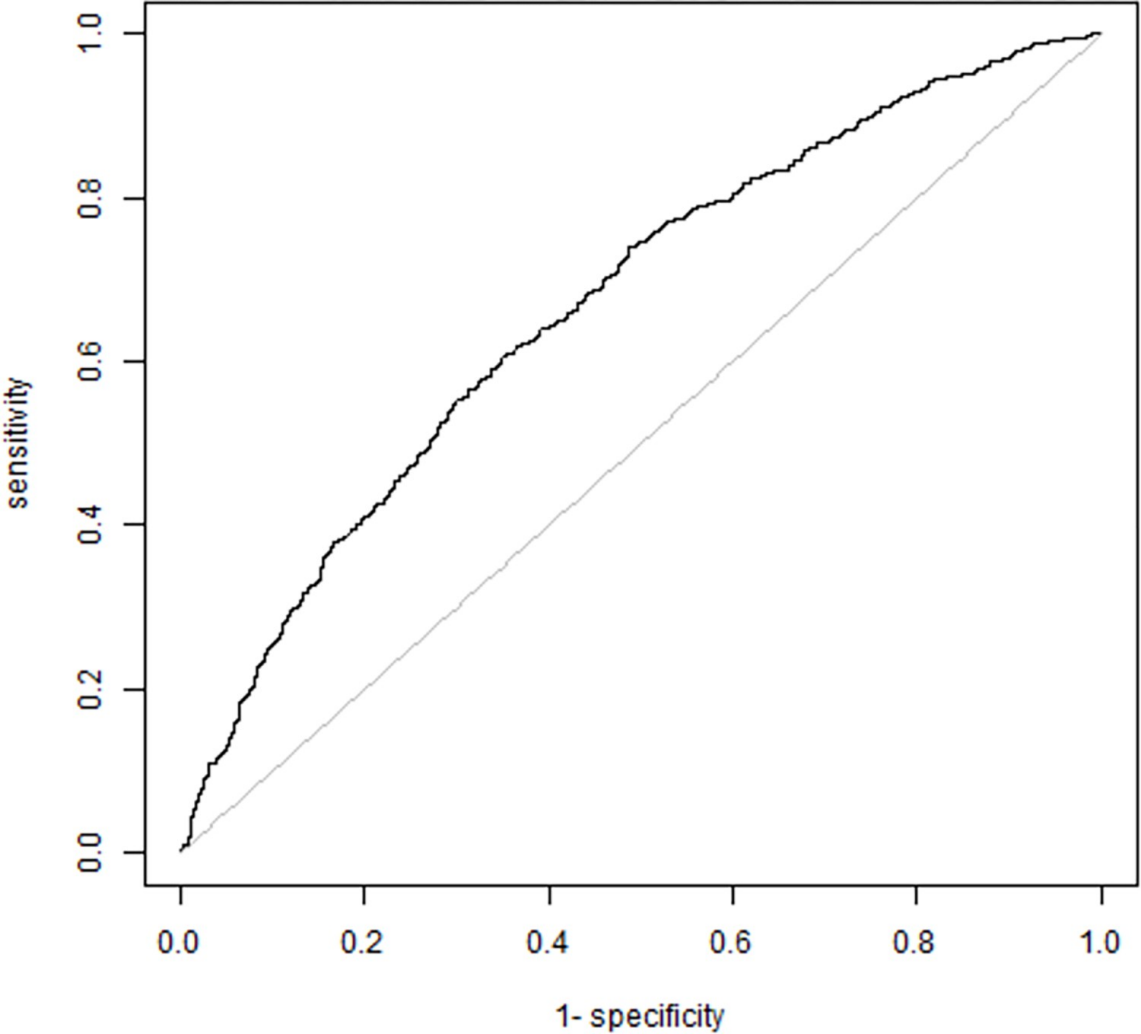
Receiver Operating Curve of composite gender score. This figure is a graphical representation of the Receiver Operating Curve presenting the quality of the prediction of BAS using our gender score.

This method is a replication of the one used by Pelletier *et al*. [36]. In the final model, participants’ predicted scores of BAS (0 = female, 1 = male) were the ones extracted as gender index scores. The predictive quality of the resulting score was then estimated using a ROC curve and its AUC (Fig 2). In this final procedure, we used an exploratory strategy. The selection of our final model is detailed in the results and discussion sections.

## Results

As evoked in the methods section, in order to select the measures to include in the EFA, we conducted several preliminary analyses. These analyses aimed to identify the more polarized variables between males and females that would be the most promising to result in a stable factorial structure, and a relevant gender score.

### Preliminary analysis

As outlined in the methods section, several preliminary t-tests and χ^2^ were performed. Preliminary analyses are presented in Tables 1 and 2. Among the variables that showed sex differences, eight of these variables composed the final model. These variables included: (1) hostile behavior during childhood and adulthood, (2) having at least a secondary school diploma or a bachelor’s degree, (3) sleep satisfaction and sleep efficacity, (4) having private housing, (5) having experienced violence during childhood, (6) having income from self-employment or autonomous work, (7) being in a common-law union, and (8) being employed. The final model excluded variables concerning the autonomous work income (MSA = 0.54), being in a common-law union (MSA = 0.53), and being employed (MSA = 0.61) due to their low MSA values and inadequate loadings making it impossible to find a stable model.

**Table 1:** Preliminary analysis results for continuous variables comparing birth-assigned males and females.

	N	Mean (SD)	M_males_	M_females_	*t*	*p*
**SHQ sleep efficacy**	1987	88.30 (18.84)	89.29 (18.32)	86.78 (19.54)	-2.866	0.004
**Depressive symptoms**	2037	12.59 (8.05)	11.47 (7.79)	14.31 (8.14)	7.814	<0.001
**Suicidal thoughts/attempts**	1214	3.02 (1.84)	2.87 (1.77)	3.26 (1.91)	3.488	0.001
**Past year suicide attempts**	1214	2.45 (1.60)	2.36 (1.56)	2.60 (1.65)	2.449	0.015
**Ever talked about suicide**	1214	2.07 (1.42)	1.96 (1.36)	2.24 (1.50)	3.249	0.001
**Suicidal risk**	1214	2.63 (1.88)	2.49 (1.80)	2.87 (1.98)	3.387	0.001
**Psychotic symptoms**	1988	0.91 (1.22)	0.92 (1.22)	0.90 (1.22)	-0.295	0.768
**Childhood hostile behavior**	2008	4.88 (5.63)	5.55 (5.80)	3.85 (5.17)	-6.887	<0.001
**Youth hostile behavior**	2008	5.91 (6.29)	6.54 (6.44)	4.92 (5.93)	-5.81	<0.001
**Teen hostile behavior**	2006	5.69 (6.32)	6.39 (6.53)	4.59 (5.81)	-6.467	<0.001
**Adult hostile behavior**	2015	7.05 (6.37)	7.53 (6.48)	6.31 (6.11)	-4.232	<0.001
**Alcohol consumption**	1998	6.36 (8.64)	7.07 (9.05)	5.26 (7.82)	-4.738	<0.001
**Substance use**	1993	2.40 (2.87)	2.90 (3.01)	1.60 (2.44)	-10.62	<0.001
**Social functioning**	1977	2.23 (1.73)	2.04 (1.64)	2.5411 (1.82)	6.209	<0.001
**Physical violence childhood**	1943	2.27 (2.49)	2.31 (2.46)	2.20 (2.54)	-0.878	0.384
**Physical abuse childhood**	1933	1.80 (2.02)	1.86 (2.00)	1.72 (2.05)	-1.48	0.139
**Sexual violence childhood**	1907	0.92 (1.58)	0.71 (1.44)	1.25 (1.73)	7.055	<0.001
**Total violence in childhood**	1947	3.18 (3.42)	3.01 (3.26)	3.45 (3.65)	2.712	0.007
**Negative urgency**	2042	10.39 (3.48)	10.24 (3.42)	10.62 (3.55)	2.409	0.016
**Positive urgency**	2041	10.76 (3.10)	10.63 (3.05)	10.95 (3.17)	2.242	0.025
**Lack of premeditation**	2041	7.51 (2.85)	7.35 (2.75)	7.76 (2.98)	3.08	0.002
**Lack of perseveration**	2043	7.48 (2.99)	7.40 (2.92)	7.60 (3.08)	1.455	0.146
**Sensation seeking**	2041	9.82 (3.38)	10.14 (3.25)	9.33 (3.52)	-5.276	<0.001
**Anxiety symptoms**	2059	50.03 (16.70)	48.05 (16.28)	53.10 (16.86)	6.736	<0.001

**Table 2:** Preliminary analysis results for categorical variables comparing birth-assigned males and females.

Variable name	Response choices	N	Males more represented	Females more represented	No difference	χ^2^	*p*
**Marital status**	5	2051	Never married	married, divorced, widow	Separated	59.763	<0.001
**In a common law union**	2	2026	No	Yes		20.637	<0.001
**Having children**	2	687	No	Yes		25.585	<0.001
**Highest primary or secondary study level completed**	3	2041	Secondary 2 or less, Secondary 3 or 4	Secondary 5		17.713	<0.001
**Highest diploma**	7	1939	< Secondary 5	Bachelor’s degree	Secondary diploma, professional diploma, college degree, university certification	25.934	<0.001
**Currently employed**	2	2054	No	Yes		3.885	0.049
**Working time schedule**	3	1943		Regular part-time	Full time, occasional part time	8.427	0.015
**If not currently employed, previous job remuneration**	2	692	Yes	No		5.3	0.021
**Income source**	15	2005	Autonomous work	Public retirement pension, Child Tax Credit, spousal support	Salary, dividends, employment insurance, worker’s compensation, employer’s retirement pension, Registered Retirement Savings Plan (RRSP), social welfare, child alimony, none, other	33.69	0.002
**Persons living with participant**	7	2054	Parents, other family members, alone	Partner, children	Other patients, other persons	121.344	<0.001
**Sleep satisfaction**	2	2061	Yes	No		37.935	<0.001
**Type of housing**	13	2051	Rooming house, homeless, other (e.g., hotel)	Private housing, social housing,	Cooperative housing, supervised apartment, guest house, group home, hospital, jail, judiciary hospital	48.878	<0.001
**Tobacco use**	5	1835	Smoking everyday	Never smokes	Smoking occasionally, don’t know/ refuse to answer	34.567	<0.001

Note: Only the significant variables were presented to reduce the size of the table.

To summarize, males tended to report higher levels of hostile behavior during childhood and adulthood (*t* = -6.89, *p*<0.001; *t* = -4.232, *p*<0.001) and were more likely to report better sleep efficiency (t = -2.87, *p* = 0.004) than females. Males were also less likely to have completed secondary education (χ^2^ = 25.93, *p*<0.001), less likely to be employed (χ^2^ = 3.88, *p* = 0.049), tended to report more often having income from autonomous work (χ^2^ = 33.69, *p* = 0.002), were less likely to have ever been married (χ^2^ = 59.76, *p*<0.001), and were more likely to be satisfied with their sleep quality (χ^2^ = 37.93, *p*<0.001) than females. Males also smoked daily more often than females (χ^2^ = 34.57, *p*<0.001). Conversely, females tended to report more violence experienced during childhood (*t* = 7.05, *p*<0.001), were more likely to have attained a bachelor’s degree (χ^2^ = 25.93, *p*<0.001), to have access to private housing (χ^2^ = 49.50, *p*<0.001), to have a stable part time job (χ^2^ = 8.43, *p* = 0.015), and were more often in a common-law union (χ^2^ = 20.64, *p*<0.001) or in a current or past married status (i.e., currently married, divorced or widowed) (χ^2^ = 59.76, *p*<0.001) than males. Males and females did not differ regarding their perceived importance of spirituality (*t* = -1.38, *p* = 0.168), or their perception of spirituality as a source of strength (*t* = -1.30, *p* = 0.193).

### Exploratory factor analysis (EFA) and logistic regression between factors and sex

#### EFA four factor model

For the first third of the sample (i.e., 33%), a four-factor model was produced comprised of eight variables, with two variables in each factor. The KMO for this model was 0.54 and the Bartlett’s sphericity test was highly significant (χ^2^(df) = 453.36 (28), *p*<0.001). The factor loadings ranged between -0.28 and 0.90, which approximates the superior |0.30| limit that allows for the emergence of a stable model. Furthermore, factor loadings under the |0.30| limit can be accepted if most of the other loadings are high enough [69].

The four-factors are composed as follows: (1) Factor 1 is composed of both hostile behavior during childhood and adulthood; (2) Factor 2 is composed of the attainment of a secondary school diploma or bachelor’s degree; (3) Factor 3 is composed of sleep efficiency and sleep satisfaction; and finally, (4) Factor 4 is composed of having private housing along with the experience of violence during childhood. Factors 1 and 3 significantly predicted being assigned male at birth (*p*<0.001). By contrast, Factor 4 did not manage to significantly predict male BAS (*p* = 0.57) and Factor 2 did not predict female BAS (*p* = 0.18). The AUC for this model is 0.61, which is considered superior to random prediction (0.5).

This model was deemed acceptable after the testing of another five-factor model composed of 11 variables that did not pass the CFA quality controls. This was due to the fifth factor not being able to significantly predict any BAS nor improve the AUC. Therefore, we removed the fifth factor and tested a final four-factor model which resulted in acceptable results in CFA. The final EFA, although not perfect, resulted in a stable model with a larger sample (n = 562 vs. n = 504) and was therefore deemed acceptable by our research team.

### Confirmatory factor analysis (CFA) and logistic regression results

#### Four-factor model CFA for validation

A CFA using the same factorial structure was then performed on two-thirds of the sample (66%). This model showed acceptable fitness measures with CFI = 0.981, RMSEA = 0.033 and SRMR = 0.025. Factors 1 and 4 significantly predicted being assigned male at birth (*p*<0.001) and Factors 2 and 3 being assigned female (*p*<0.001). The AUC for this model was 0.66, which is superior to random prediction.

#### Final CFA on the whole sample

The final four-factor model developed using the whole sample is presented in Table 3. It showed acceptable fitness with CFI = 0.966, RMSEA = 0.044, SRMR = 0.030. Factors 1 and 4 significantly predicted being assigned male at birth (*p*<0.001) and Factors 2 and 3 being assigned female at birth (*p*≤0.001). The AUC for this model is 0.67 (visually presented in Fig 2), which is superior to the AUCs of both previous models with subsamples.

**Table 3:** Factorial composition of the final gender score, factor loadings, and logistic regression results for BAS.

	Factor 1	Factor 2	Factor 3	Factor 4
**Hostile behavior during childhood**	0.767			
**Hostile behavior during adulthood**	0.835			
**Secondary diploma**		0.494		
**Bachelor’s degree**		0.438		
**Sleep satisfaction**			-0.795	
**Sleep efficacy**			-0.425	
**Private housing**				0.114
**Sexual violence during childhood**				-0.918
**Factors regressions with BAS; OR>1 = male sex, OR<1 = female sex**	OR = 1.63 *p*<0.001	OR = 0.75 *p* = 0.001	OR = 0.70 *p*<0.001	OR = 1.63 *p*<0.001

Note: N = 1708, χ2 = 61.337; df = 14; *p*<0.001; CFI = 0.966; RMSEA = 0.044, SRMR = 0.030.

**Fig 3:**
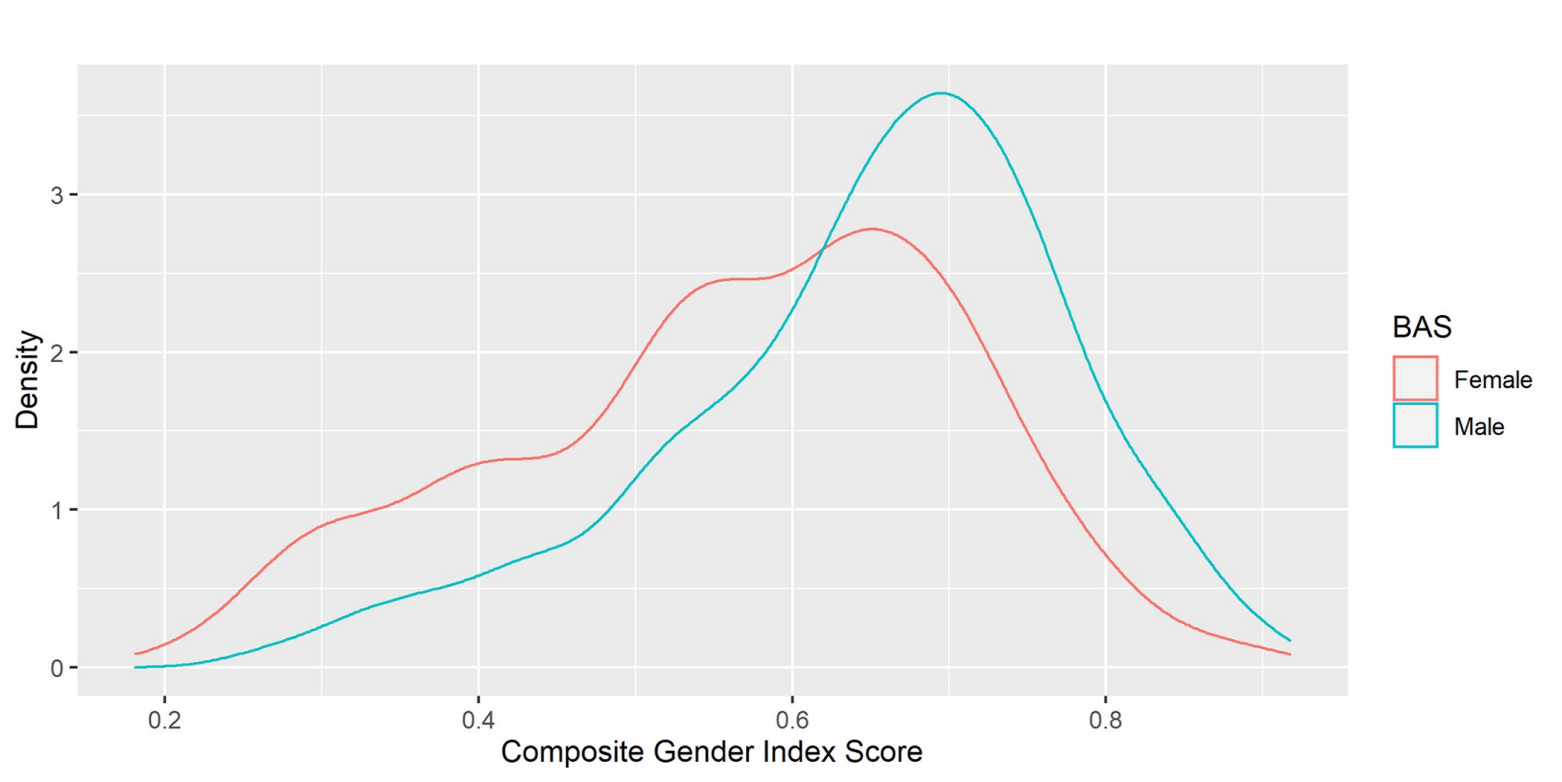
Gender score repartition depending on birth-assigned sex. This figure presents visually the repartition of gender score depending on the birth assigned sex of our participants.

This AUC indicates a partial overlap between sex and gender using our composite measure. Fig 3 shows the distribution of the final composite gender index score as a function of BAS. This figure indicates that females tend to have a gender score that is more spread out along the continuum than males. This distribution is similar to the results identified by Pelletier et al. [36].

### New analysis with the gender index and comparison with the analysis conducted by sex

To verify and replicate the differences in associations among psychiatric symptoms as explained by BAS versus the composite gender index, we conducted several analyses. First, we conducted correlational analyses between BAS, gender scores, and psychiatric symptoms to ascertain an effect magnitude comparison. Then based on the distribution, the gender index scores were split in terciles to create groups that were classified as masculine, feminine, or undifferentiated to allow for group comparisons with BAS. This is possible given the sufficient power provided by the Signature sample size. We conducted ANOVAs using these terciles as grouping variables to compare symptom scores. These ANOVAs were compared with t-tests conducted with sex as a grouping variable. Then, we evaluated the diagnosis repartition depending on BAS and gender terciles created earlier using χ^2^. Finally, we created groups pairing BAS and gender terciles to create six groups to investigate the intertwining effects of sex and gender in our dataset.

#### Correlations between gender score, birth-assigned sex, and psychiatric symptoms

Correlations were performed to identify the magnitude of relations between BAS, the composite gender score, and psychiatric symptoms. Results are presented in Table 4.

**Table 4:** Correlations between composite gender score, birth-assigned sex (BAS) and variables of interest.

	BAS	Negative urgency	Positive urgency	Lack of premeditation	Lack of perseveration	Sensation seeking
**BAS**	1.000	-0.042	-0.032	-0.053*	-0.005	0.110***
**Gender score**	0.289***	0.105***	0.119***	0.100***	0.065**	0.210***
**BAS**	**Depressive symptoms**	**Anxiety**	**Alcohol use**	**Drug use**	**Deficit scale**	**Psychotic symptoms**
**Gender score**	-0.155***	-0.132***	0.107***	0.221***	-0.135***	0.002

Note

* = *p*<0.05

** = *p*<0.01

*** = *p*<0.001; N = 1708; Pearson correlations used; BAS coded as 0 = female and 1 = male. Therefore, positive coefficients mean males have higher scores, for example.

Compared to females, males reported sensation seeking more often (*r* = 0.110; *p*<0.001) as well as using more alcohol (*r* = 0.107; *p*<0.001) and drugs (*r* = 0.221; *p*<0.001). On the other hand, females reported more depressive (*r* = -0.155; *p*<0.001) and anxious (*r* = -0.132; *p*<0.001) symptoms and report lacking premeditation more often (*r* = -0.053; *p*<0.05) than males. Furthermore, females in our sample reported having more functional difficulties (*r* = -0.135; *p*<0.001) than males.

We then investigated relations between symptoms and the created gender score. First, this score was related to BAS in a positive small to moderate association (*r* = 0.289; *p*<0.001), indicating a relatively moderate overlap in these concepts as presented earlier with our AUC. Second, a masculine score was related to more negative urgency (*r* = 0.105; *p*<0.001), positive urgency (*r* = 0.119; *p*<0.001), lack of premeditation (*r* = 0.100; *p*<0.001), lack of perseverance (*r* = 0.065; *p*<0.001), and sensation seeking (*r* = 0.210; *p*<0.001). Furthermore, a feminine gender score was related to more anxious (*r* = -0.144; *p*<0.001), and depressive symptoms (*r* = -0.156; *p*<0.001), and general functional deficit (*r* = -0.126; *p*<0.001). Lack of premeditation was negatively correlated to BAS but positively correlated with our gender score. Psychotic symptoms did not significantly correlate with BAS nor the gender score in our sample.

#### ANOVAs vs. t-tests

Using point-biserial correlations (i.e., using a dichotomous variable for correlations) such as what we did in the previous section to investigate the potential relations between BAS and psychiatric symptoms has limitations that we now address. In order to have comparability between a continuous and a dichotomous variable, it is possible to create groups depending on the score of the continuous variable. In this way, we can compare gradients using similar analyses to have a global portrait of the relations between our created gender score, BAS, and psychiatric symptoms in a correlative perspective and mean difference.

In accordance, we split our gender score in three groups using the tercile method to have groups of similar size. Our total group showed a mean gender score of 0.62, with males (as split by BAS) presenting a score of 0.65, while females had a score of 0.56. The tercile groups showed mean gender scores of 0.76 for the masculine group, 0.64 for the undifferentiated group, and 0.45 for the feminine group. Out of the 1,053 males of our final sample (n = 1708), 435 (41.3%) formed the masculine group, 365 formed the undifferentiated group (34.7%), and 253 formed the feminine group (24%). Regarding females, out of the 655 in our sample, 134 formed the masculine group (20.5%), 204 formed the undifferentiated group (31.1%), and 317 formed the feminine group (48.4%).

Our t-test results show similar tendencies to the ones observed with correlations. No significant differences were observed using BAS with variables measuring impulsivity, except for sensation seeking (*t* = -4.51; *p*<0.001) where males showed higher scores, and lack of premeditation (*t* = 2.18, *p*<0.05) where females showed higher mean scores than males. Similarly to the correlation results, females showed higher mean scores for depressive (*t* = 6.40; *p*<0.001) and anxious symptoms (*t* = 5.51; *p*<0.001), as well as general functional deficit (*t* = 5.47; *p*<0.001). On the other hand, males showed higher mean scores for alcohol (*t* = -4.59; *p*<0.001) and substance use (*t* = -9.85; *p*<0.001). No significant difference for psychotic symptoms between males and females was observed (*t* = -0.10; *p* = 0.921).

ANOVAs using our three gender groups offered a different perspective on BAS-based results. First, our masculine group showed higher mean scores than the two other groups on every subscale measuring impulsivity, whether being negative urgency (F = 10.54; *p*<0.001) or positive urgency (F = 14.51; *p*<0.001), lack of premeditation (F = 9.32; *p*<0.001), perseverance (F = 4.78; *p*<0.001), and sensation seeking (F = 39.96; *p*<0.001). This masculine group also showed higher levels of alcohol (F = 15.39; *p*<0.001) and substance use (F = 65.02; *p*<0.001) than the other groups. Second, our feminine group showed higher mean scores on depressive (F = 23.86; *p*<0.001) and anxious symptoms (F = 19.98; *p*<0.001), but also general functional deficit (F = 11.8; *p*<0.001) than the other groups. Interestingly, our undifferentiated group is similar to the feminine group regarding impulsivity measures, along with drug and alcohol consumption (i.e., significantly lower than the masculine group), but it is similar to the masculine group on anxious and depressive symptoms and general deficit scale (i.e., significantly lower than the feminine group). There is no significant difference on psychotic symptoms between our gender groups. All these results are presented in Table 5.

**Table 5:** ANOVAs using grouped gender score compared to t-tests using BAS.

	N	Analysis type	General mean	Masculine or male mean	Undifferentiated mean	Feminine or female mean	F/*t* (df)	*p*
**Gender score**	1708	N/A	0.62	Masculine = 0.76, Male = 0.65	0.64	Feminine = 0.45, Female = 0.56	N/A	N/A
**Negative** **urgency**	1708	ANOVA	10.31	10.84 ^a,b^	9.97 ^a^***	10.11 ^b^***	10.54 (2, 1705)	<0.001
		t-test		10.19	N/A	10.49	1.749 (1706)	0.0804
**Positive** **urgency**	1708	ANOVA	10.67	11.23 ^a,b^	10.42 ^a^***	10.35 ^b^***	14.51 (2, 1705)	<0.001
		t-test		10.59	N/A	10.79	1.30 (1706)	0.1922
**Lack of** **premeditation**	1708	ANOVA	7.48	7.90 ^a,b^	7.29 ^a^***	7.25 ^b^***	8.44 (2, 1131.6)	<0.001
		t-test		7.36*	N/A	7.67*	2.18 (1313.8)	<0.05
**Lack of** **perseverance**	1708	ANOVA	7.44	7.75 ^a,b^	7.24 ^a^**	7.33 ^b^*	4.78 (2, 1705)	<0.01
		t-test		7.43	N/A	7.46	0.21 (1706)	0.8316
**Sensation seeking**	1708	ANOVA	9.79	10.79 ^a,b^	9.41 ^a^***	9.16 ^b^***	39.96 (2, 1705)	<0.001
		t-test		10.082***	N/A	9.31***	-4.51 (1310)	<0.001
**Depression**	1708	ANOVA	12.35	10.99 ^a^***	11.89 ^b^***	14.15 ^a,b^	23.86 (2, 1705)	<0.001
		t-test		11.36	N/A	13.93	6.40 (1329.2)	<0.001
**Anxiety**	1708	ANOVA	49.83	47.69 ^a^***	48.38 ^b^***	53.42 ^a,b^	19.98 (2, 1705)	<0.001
		t-test		48.07	N/A	52.67	5.51 (1706)	<0.001
**Alcohol**	1707	ANOVA	6.27	7.84 ^a,b^	5.72 ^a^***	5.25 ^b^***	13.99 (2, 1129.4)	<0.001
		t-test		6.98	N/A	5.12	-4.59 (1532.2)	<0.001
**Drug**	1707	ANOVA	2.35	3.40 ^a,b^	2.02 ^a^***	1.65 ^b^***	57.56 (2, 1125.2)	<0.001
		t-test		2.85	N/A	1.56	-9.85 (1609.2)	<0.001
**Deficit scale**	1699	ANOVA	2.19	1.97 ^a^***	2.14 ^b^**	2.46 ^a,b^	11.72 (2, 1129.3)	<0.001
		t-test		2.01	N/A	2.48	5.47 (1257.8)	<0.001
**Psychotic symptoms**	1679	ANOVA	0.87	0.92	0.85	0.83	0.892 (2, 1705)	0.41
		t-test		0.87	N/A	0.86	-0.10 (1677)	0.921

Note: Letters are used to indicate the significant differences determined using post-hoc analyses. Therefore, groups having the same letter significantly differ from one another. Significance thresholds for post-hoc analyses are added next to the letter indicating the relation and are presented as follows

* = *p*≤0.05

** = *p*≤0.01

*** = *p*≤0.001.

#### Chi-squares to evaluate diagnosis repartition between sex and gender score

We then checked diagnosis rates depending on BAS and created gender groups. Results are presented in Table 6. In our sample, males were more often diagnosed with psychotic disorders and substance use disorders than females. Conversely, females were more often diagnosed with mood and personality disorders than males. There was no significant difference between birth-assigned sexes regarding anxious disorder diagnostic frequencies in our sample.

**Table 6:** Diagnosis repartition depending on birth-assigned sex (BAS) and gender score.

Diagnostic categories	Male BAS	Female BAS	Masculine gender	Undifferentiated gender	Feminine gender
**Psychotic disorders and schizophrenia**	462 ^a^ (44.9%)	204 (31.6%)	232 ^a^ (41.4%)	242 ^a^ (43.7%)	192 (34.3%)
**Anxious disorders, OCD, and PTSD**	104 (10.1%)	62 (9.6%)	45 (8%)	51 (9.2%)	70 ^a^ (12.5%)
**Mood disorders**	275 (26.8%)	248 ^a^ (38.4%)	141 (25.1%)	167 (30.1%)	215 ^a^ (38.5%)
**Personality disorders**	76 (7.4%)	111 ^a^ (17.2%)	76 (13.5%)	55 (9.9%)	56 (10%)
**Substance use disorders**	111 ^a^ (10.8%)	21 (3.3%)	67 ^a^ (11.9%)	39 (7%)	26 (4.7%)
**Total**	1028	646	561	554	559
	1674		1674		

Note: Letters are used to indicate the significant differences determined using post-hoc analyses. Therefore, groups having the same letter significantly differ from one another. χ^2^ by BAS = 97.8, *p*<0.001; χ^2^ by gender score = 52.9, *p*<0.001.

When looking at the diagnosis repartition depending on gender groups, we could observe that the masculine and undifferentiated groups were more often diagnosed with psychotic disorders than the feminine group. The feminine group was more often diagnosed with mood and anxious disorders than the others. Finally, the masculine group was more often diagnosed with substance use disorder than the others. There was no difference in personality disorder frequency of diagnosis regarding gender groups.

#### ANOVAs with birth-assigned sex and gender interaction on psychiatric symptoms

**Fig 4:**
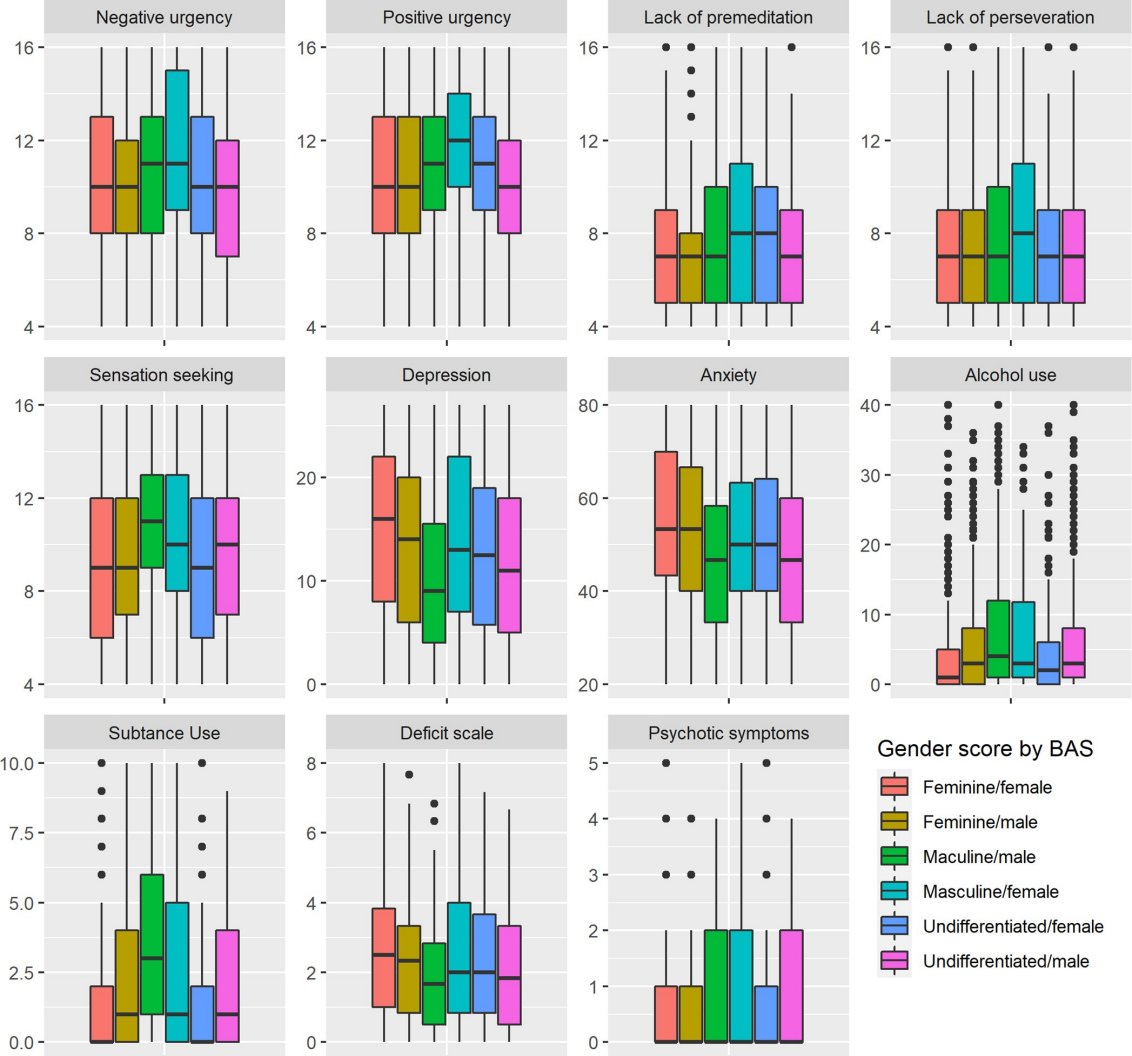
Scores depending on groups representing BAS and gender scores. This figure graphically represents the scores of our groups crossing gender score and BAS on impulsivity measures and psychiatric symptoms.

We then created groups crossing BAS and gender to investigate their combined influence on psychiatric symptoms. These groups are therefore reflecting persons of both BASs distributed across our three gender groups, resulting in six groups. We can observe several statistically significant differences between these groups. These results are presented in Table 7 and visually represented in Fig 4.

**Table 7:** ANOVA comparisons when pairing birth-assigned sex (BAS) and gender score terciles.

	General mean	F/F mean	F/M mean	M/M mean	M/F mean	U/F mean	U/M mean	F	*p*
**Negative urgency**	10.31	10.16 ^a^*	10.04 ^b^*	10.68 ^d^*	11.38 ^a,b,c^	10.43	9.72 ^c^**^, d^*	6.226	<0.001
**Positive urgency**	10.67	10.39 ^a^**^, e^	10.29 ^b^**^, f^	11.07 ^e,f,g^	11.78 ^a,b,c,d^	10.77 ^c^	10.23 ^d^**^, g^*	7.749	<0.001
**Lack of premeditation**	7.48	7.31 ^a^**	7.17 ^b^**	7.69 ^c,f^	8.59 ^a,b,c,d,e^	7.64 ^d^	7.10 ^e^**^, f^	6.871	<0.001
**Lack of perseveration**	7.44	7.22 ^a^	7.48	7.61	8.22 ^a, b^*	7.34	7.19 ^b^*	3.103	0.009
**Sensation seeking**	9.79	8.98 ^a^**^, e^**	9.39 ^b^**^, f^	10.87 ^a,b,c,d^	10.53 ^e,f,g^	9.03 ^c^**^, g^**	9.62 ^d^**	17,51	<0.001
**Depression**	12.35	14.86 ^a^**^, e, f^**	13.25 ^b^**	10.16 ^a,b,c,d^	13.69 ^c^**	12.63 ^d^*^, e^	11.48 ^f^**	15.54	<0.001
**Anxiety**	49.83	54.09 ^a^**^, d^**	52.58 ^b^**^, e^**	46.71 ^a,b,c^	50.87	51.63 ^c^*^, f^*	46.57 ^d,e,f^*	12,02	<0.001
**Alcohol**	6.27	4.52 ^a^**^, b^	6.16	7.99 ^a^**^, c^**	7.35 ^b,d^	4.58 ^c^**^, d^	6.35	8.555	<0.001
**Drug**	2.35	1.28 ^a^**^, f^*^, g, h^	2.10 ^b^**^, f^*^, k^	3.62 ^a,b,c,d,e^	2.68 ^c^*^, g^**^, i^**	1.26 ^d^**^, i,j,k^	2.44 ^e^**^, h^**^, j^**	36.83	<0.001
**General deficit scale**	2.19	2.59 ^a^**^, e^**	2.29 ^b^*	1.83 ^a,b,c,d^	2.44 ^c^*	2.35 ^d^*	2.03 ^e^**	9,269	<0.001
**Psychotic symptoms**	0.87	0.83	0.82	0.89	1.04	0.80	0.88	0.804	0.547

Note: F/F = Feminine Female, F/M = Feminine Male, M/M = Masculine Male, M/F = Masculine Female, U/F = Undifferentiated Female, U/M = Undifferentiated Male.

N size: F/F = 317, F/M = 253, M/M = 435, M/F = 134, U/F = 204, U/M = 365.

Letters are used to indicate the significant differences determined using post-hoc analyses. Therefore, groups having the same letter significantly differ from one another. Significance thresholds for post-hoc analyses are added next to the letter indicating the relation and are presented as follows: Letter(s) only = *p*≤0.05

* = *p*≤0.01

** = *p*≤0.001.

Regarding negative urgency (F = 6.226; *p*<0.001), we can observe that masculine females show the higher scores of our sample (11.38), and a significantly higher mean score than feminine females (10.16), feminine males (10.04), and undifferentiated males (9.72). Close by are masculine males that show a significantly higher score (10.68) than undifferentiated males (9.72).

Positive urgency shows a similar tendency (F = 7.749; *p*<0.001) with masculine females showing the highest mean score of the sample (11.78), significantly higher than feminine females (10.39), feminine males (10.29), undifferentiated females (10.77), and undifferentiated males (10.23). Masculine males show the second highest mean score (11.07), that is significantly higher than feminine females, feminine males, and undifferentiated males.

Lack of premeditation follows the same trend (F = 6.871; *p*<0.001), with masculine females showing the highest mean score of the sample (8.59), which is significantly higher than all the others. Masculine males show the second highest mean score (7.69) of the sample, which is significantly higher than undifferentiated males (7.10).

Regarding lack of perseverance, masculine females again show the highest score (8.22), which is significantly higher (F = 3.103; *p* = 0.009) than feminine females (7.22), and undifferentiated males (7.19).

Sensation seeking shows a slightly different trend with masculine males having the highest mean score of all (10.87), which is statistically higher than feminine females (8.98), feminine males (9.39), undifferentiated males (9.62), and females (9.03). Masculine females also show a significantly higher mean score (10.53) than feminine females, feminine males, and undifferentiated females.

For depressive symptoms scores, the trend is reversed. Feminine females show the highest mean score of all (14.86) which is significantly higher than masculine males (10.16), undifferentiated females (12.63), and undifferentiated males (11.48). On the other hand, masculine males show the lowest mean score of all, which is significantly lower than the others, except undifferentiated males.

A similar trend is observed with anxious symptoms, with feminine females showing the highest score of all (54.09), while masculine males and undifferentiated males show the lowest mean scores (46.71 and 46.57 respectively). Both groups show significantly lower scores than feminine females, feminine males (52.58), and undifferentiated females (51.63).

Regarding alcohol consumption, we can observe a significant difference (F = 8.55; *p*<0.001) driven by masculine males displaying the highest mean score (7.99), which is significantly higher than the one for feminine females (4.52) and undifferentiated females (4.58). This significant difference is also driven by masculine females, that have the second highest mean score (7.35), which is also significantly higher than feminine females, and undifferentiated females.

Regarding drug use, masculine males show the highest score of all (3.62) which drives the observed significant difference (F = 36.83, *p*<0.001), due to its statistically significant difference with all other groups, and also undifferentiated and feminine females that present the lowest scores of the groups (1.26 and 1.28 respectively) which is significantly lower than the other groups.

Regarding general deficit, we can observe a significant difference (F = 9.269; *p*<0.001) driven by masculine males showing the lowest mean score (1.83), which is significantly lower than other groups, except for undifferentiated males (2.03) which have a significantly lower score than feminine females (2.59).

## Discussion

Through a trial-and-error validation method, we constructed a composite gender index score reflecting the indirect gender-based traits of our participants emerging from the data collected. This gender index was built using eight variables: (1) hostile behavior during childhood and/or (2) adulthood, (3) secondary school diploma and/or (4) bachelor’s degree, (5) sleep satisfaction, (6) sleep efficiency, (7) access to a private housing, and (8) childhood experiences of sexual violence, as presented in Table 3. This composition resulted in a stable factorial structure with all factor loadings >|0.4| (except for private housing), good fit indices, and a sufficiently powered sample size, which made this final model acceptable. This is an important step towards applying similar approaches to psychiatric research as well as other related disciplines (e.g., psychology, neurosciences) that may not have included direct measurement of sociocultural gender variables.

Our composite score allowed an acceptable, although imperfect, prediction of BAS with 67% accuracy. This modest prediction is still relevant in our case, as a perfect prediction of BAS would not bring much added value. The goal of the composite gender index was not to perfectly predict BAS, but to indirectly measure sociocultural gender. As postulated by Pelletier *et al*. [36], the AUC of a similar composite gender index identifies an overlap between sex and gender. These concepts are intertwined but do not necessarily overlap perfectly. Indeed, propensities towards masculinity and femininity can be considered as individual psychosocial characteristics that reflect life experiences and also social, cultural, and behavioral aspects of these individuals’ gender that can be distinct from BAS [70].

While sociocultural gender can be independent of one’s BAS, there is a certain overlap between observable features of this identity and the socially reinforced stereotypes and gender norms that are often oppressively at play. The partial overlap of these constructs is also confirmed by the moderate correlation observed between BAS and our composite gender score (*r* = 0.29; *p*<0.001). It is nonetheless important to underline that our composite gender score represents gendered lived experiences rather than gender identity *per se*. However, a more complete measure of gender is composed of several aspects that include gender identity and gender roles that are further shaped by unmeasured ties to gender relations and institutionalized gender [18].

Few studies to date have evaluated gender among psychiatric patients. Nonetheless, a study conducted in 1982 in the United States measuring gender roles in a psychiatric sample and in college students identified a similar distribution of gender among both populations [71]. Although gender is a concept that evolves across time and locations, this result can support the comparability of a gender measure across psychiatric and non-psychiatric samples. Furthermore, as presented in Fig 3, the gender score repartition follows a similar pattern to the one presented in Pelletier *et al*.’s work. Similarly, females tend to have a score that is more spread out along the continuum, while males tend to be more grouped around a masculine score. The functional significance of this will need to be further explored.

In summary, our hypotheses are confirmed. First, we hypothesized that our composite gender score would allow for the identification of statistical differences regarding psychiatric symptom severity. This hypothesis was confirmed by our correlations and ANOVAs conducted using our three gender groups based on terciles (feminine, undifferentiated, masculine). The correlations also highlight that our gender score allows for the identification of differences that are absent when using BAS alone, especially for some impulsivity measures. Indeed, these associations seem to be of a slightly higher effect size, except for the general deficit scale from the World Health Organization Disability Assessment Schedule (WHODAS). This scale has a slightly more powerful association with BAS (*r* = -0.135) than our gender score (*r* = -0.126).

Regarding psychiatric diagnosis, we confirmed our second hypothesis stipulating that sex and gender repartition will change depending on the disorders. We can argue that some disorders seem sexed while other seem gendered. When looking more closely at the observed discrepancies in the relations between our gender score, BAS, and psychiatric symptoms, we can observe some patterns that partially corroborate the literature, but also deepen our understanding of how gender relates with those symptoms among a population that is often neglected in such research domains.

Impulsivity, for example, is identified in many psychiatric disorders such as substance use disorder [72,73], eating disorders [73], or bipolar disorder [74]. Regarding alcohol use disorder, for instance, lifetime prevalence revolves around 30% in the United States and Australia [75], and fluctuates in other countries [76]. In Canada, overall lifetime alcohol abuse and dependence was reported to be 18.1% in 2012, while being more common for males (26.6%) than females (9.8%) [77], a similar tendency that is observed worldwide [75]. Another trend can be identified with cannabis, opioids, and other substances use disorders, with males being more represented than females in Canada and other countries [75,77]. Although, the gap seems to be narrowing [78], it underlines the role of sociocultural expectations between genders. For example, consumption of tobacco and alcohol diminished across time for males in France, but has risen for females [64]. These patterns are related to other social factors such as work status or educational attainment, along expected gender roles and behaviors [64].

Furthermore, the relation between BAS, gender score, and impulsivity indicated higher impulsivity among individuals with a masculine gender score. We identified significant differences regarding lack of premeditation (*t* = 2.18, *p*<0.05) and sensation seeking (*t* = -4.51, *p*<0.001) based on BAS, where males tend to show higher rates of sensation seeking, while females tend to lack premeditation more often. This interaction is related to the fact that masculine females have the highest score of the crossed groups and, therefore, masculinity shares a similar magnitude of importance with feminine sex. This in turn seems logical due to the differences in risk-taking behaviors that are observed in the literature, where males usually take more risks [79], and also due to the evolution of substance use patterns influenced by sociocultural factors [64]. This relation between impulsivity and masculinity can also be seen in our sample when looking at substance use disorders, as they seem more common in males and masculine individuals.

These results are on line with the literature regarding sensation seeking [80,81], but not regarding lack of premeditation where there is no statistically significant difference between males and females observed in the general population [80,81]. Furthermore, there are also differences identified in the literature that are not observed in our results, such as males showing higher scores of positive urgency [81] or lack of perseverance [80]. Such differences with the literature could be due to our sample, since most of the studies using the UPPS-P scale used it in a non-psychiatric context.

Unfortunately, we did not find any research measuring impulsivity while considering sociocultural gender as an independent variable. We therefore cannot compare our results to existing literature. Despite that, gendered socialization could also explain this complex relation. The social environment could have favored impulsive behavior and consumption [82], and reflect internalized gender roles and processes [83]. Indeed, masculinity is related to higher substance use and antisocial behaviors [84]. Nevertheless, as stated, «sex» and «gender» are not interchangeable terms and refer respectively to biological and sociocultural characteristics. Regarding impulsivity, such differences experienced between individuals can be due to sexual dimorphism in brain development–although such differences might be more complex than just a binary distinction as “male/female”, hormonal differences or sociocultural experiences [85]. In regard to the other measured psychiatric symptoms, as presented, the correlations with BAS and our gender score are similar in direction and magnitude, although perhaps slightly stronger with our gender score.

Lengua and Stormshak [84] developed a model evaluating the relation between BAS, gender roles, and psychological symptoms (i.e., depression, antisocial behaviors, and substance use). Although the authors report a moderate fit to their data, their model identified that BAS was not significantly related to any symptoms when controlling for gender role [84]. This indicates that gender roles may be more appropriate to explain substance use, which is partly corroborated by our results using a composite gender index. It also underlines the crucial importance of including gender measures in mental health research, but also in psychology and psychiatry at large.

Some disorders, on the other hand, are expected to be more feminine. For example, a systematic review on anxious disorders identified a prevalence in the general population fluctuating between 3.8% and 25% [86], with females more represented in a 2:1 ratio [86,87]. This result is not exactly confirmed in our results as anxious disorders seem to be as common between males and females in our sample but appear more prevalent in persons showing more feminine characteristics. One explanation would be the characteristics of the participants composing our sample. The presented literature review [86] focuses on diagnosis of anxious disorders among the general population, not in psychiatric hospital inpatients. The severity of the disorder, resulting in help seeking behavior, and hospitalization, could blur the differences observed in the general population. Furthermore, some studies investigating anxious disorder diagnoses and prevalence among specific groups, such as non-prescribed opioid users, did not find an effect of BAS [86].

When looking at specific anxious disorders–such as social anxiety disorder–it is reported that females tend to have a higher prevalence in the general population, but the opposite is observed in clinical settings [75]. Although we do not observe a difference in diagnosis rate based on BAS, we observed a significant difference based on our gender score with a 1.6:1 masculine to feminine diagnosis ratio in our sample. This indicates that differences in prevalence of anxious disorders seem to be better explained in our sample by sociocultural factors rather than biological differences between males and females.

Mood disorders follow a similar trend but are much clearly feminine in our sample, with females and feminine gendered persons being more represented in this category. We can observe a similar tendency in the literature with major depressive disorders one-year prevalence of 7% in the United States, with variations depending on age, and higher prevalence in females [75]. Females also show higher prevalence for persistent depressive disorder, and type II bipolar disorder (although results are not as robust) [75]. No significant BAS difference was observed in type I bipolar disorder and cyclothymic disorder [75].

Consequently, observed differences in prevalence rates between males and females may be related to gender roles and especially masculine gender roles. For example, as presented in the literature, depression manifests itself and is expressed differently among males and females. In general, males lack self-identification and help-seeking of health professionals. This in part explains the higher suicide rates observed among males [88]. Masculinity is also built around social norms such as individuality, independence, stoicism, autonomy, achievement, and aggressivity. This puts high pressure on males to remain tough and not show any weakness while facing adversity [88]. This pressure can result in masking and lower help-seeking behavior, explaining a part of the significant difference in prevalence rate of depression or other mood disorders. This could also explain the higher rate of substance use disorder, which could be viewed as a manifestation of distress and a coping mechanism [88].

The differences we observed in symptom severity, especially for anxiety and depression, are also observed in the literature evaluating the relative influence of gender and gender roles on these symptoms. For example, Arcand et al. [89] observed that masculinity had a protective role for anxiety and depression, while femininity was related to higher anxiety but lower depression when considering just gender roles. These results echo ours, where we can see that our gender score negatively relates to anxious and depressive symptoms, therefore indicating that higher masculinity reflects lower scores, what was confirmed with our ANOVAs. A recent meta-analysis by Lin et al. [90] also confirmed the protective role of masculinity on depression and anxiety, but also a clear evolution in time with its protective ability currently diminishing, confirming the importance of the frame surrounding these measures, whether temporal or geographical. On the other hand, it is known that female sex is related to higher prevalence of depressive and anxious disorders [91], which we corroborate as well.

The reverse phenomenon was observed regarding personality disorders, which seemed to be more common among females in our sample, but similarly distributed across our gender groups. The literature underlines a lack of research on prevalence of personality disorders due to their complex classification and difficulty to properly diagnose [92]. Nonetheless, their prevalence is estimated to be between 4 and 15%, and is as common between males and females in the general population [92]. Furthermore, as presented in the DSM-5-TR, some personality disorders are more common among males (e.g., antisocial, narcissistic, and schizotypal personality disorder), while other personality disorders are more in females (e.g., avoidant, histrionic, and dependent personality disorders) [75]. Despite that, borderline personality disorder is reported to be as common in males and females in the general population but is more prevalent among females in a clinical setting, such as ours.

It is important to underline the socially constructed nature of personality disorders [93]. Indeed, this social construction echoes strongly with the sociocultural dimension of gender. Some authors suggest that borderline personality disorder, for instance, is a gendered construct that emerges from the social construction of the psychiatric classification system [93,94]. In this regard, there is a lack of literature regarding sociocultural gender [75] that should be explored further.

In addition, psychotic disorders seem to be more masculine overall in our sample, even though their gendered repartition appears similar between masculine and undifferentiated groups. The literature shows that the prevalence of schizophrenia fluctuates between 0.7 and 1% of the population, and that males are more often diagnosed than females [28]. Nonetheless, recent literature seems to mitigate the difference in prevalence ratio between males and females, and indicate a similar prevalence between the sexes [75], especially for lifetime prevalence as females tend to be diagnosed later than males [28]. There is a general lack of literature regarding schizophrenia in relation to sociocultural gender [28], but some tendencies have been identified. Interestingly, early literature evaluating gender roles in schizophrenia patients identified reversed gender roles [28]. More recent studies have identified a more “neutral” gender role pattern in participants on both feminine and masculine subscales [28], corroborating our results. This tendency could be explained with a clinical perspective. Persons suffering from schizophrenia, while being more often males, can experience a different socialization path than persons outside of the psychotic spectrum, resulting in different identities, personality presentation, and expected social roles.

Overall, this perspective that some disorders are sexed while others are gendered is supported and partially explained by differences in psychiatric symptoms severity. The results displayed in Table 7 can be interpreted as a gradient of sex versus gender in experienced symptoms. Some differences observed can reflect that gender is more relevant, while BAS seems to be more relevant in other situations. For example, regarding general deficit, groups composed of female sex show higher scores than male sex groups. This indicates that such a symptom seems to be more sexed than gendered. A similar tendency is observed with depressive symptoms where female sex groups score higher, but feminine gender seems to play a role as well.

Moreover, impulsivity measures show a similar trend overall, with masculine gender appearing to be more important than male sex for this trait. With anxious symptoms, feminine gender seems to be more important, closely followed by female sex. With alcohol and substance use, masculine gender seems more important, followed by male sex. These results could be interpreted as an indication that gender is more important than sex regarding impulsivity, and that concordant sex and gender (i.e., masculine males and feminine females) are expected to be the main vehicle of difference between our groups.

These results can confirm that sex and gender are deeply intertwined but distinct concepts. Although we could argue that for some issues, sex seems more relevant than gender, gender does seem to be crucial for other considerations. Our results are a clear example of complexity in the context of mental health and of the relative, cumulative, and interactive importance of both concepts of sex and gender. Unfortunately, due to the small representation of gender diverse individuals in our sample, a cross-validation using gender identity is not possible with the current analysis. This underlines the need for replication of our method to confirm its validity and relevance to the concept and measurement of gender.

### Limitations

The current study includes psychiatric emergency patients and therefore people in psychological crisis. This warrants discussion with regards to methodology, reproducibility, and generalizability. The use of factorial analyses in gender research and in psychometrics at large is not a new method. Furthermore, the method we used already showed robust results in Pelletier, Ditto, and Pilote’s work [36]. Our method is inspired from this innovative approach. The main difference is our use of a data-based approach, along with a psychiatric population rather than a cardiovascular one. Another limitation is the predictive capacity of our gender score, which is modest at best. Nonetheless, this score seems to be measuring gendered lived experiences or some latent gender construct. This procedure is the first to use this specific method and will need to be replicated to confirm its quality and adaptability to other disciplines beyond psychiatry.

Nonetheless, this method also limits the comparability of results between studies. Variables used in our protocol reflect what was available in our dataset, which can majorly differ from other datasets. This method calls for researchers to create their own gender scores using available data that can vary in quantity, but also qualitatively regarding sex-related representativity. This method also might not perfectly represent gender in its totality and complexity, and other relevant measures constructed specifically for this purpose should be used in novel research projects such as the Stanford GHVR survey [95], for example. Despite this limitation, we could argue that compared to Pelletier et al.’s or Smith and Koehoorn’s work, our method allowed the emergence of a stable model with a limited number of gendered variables, underlying the flexibility and relevance of this method in the specific secondary data use.

As our population studied in this protocol is very specific, the generalizability of our results regarding symptom severity and rates of diagnoses are limited to this group. Despite that, as evoked earlier in the discussion, gender roles seem to be distributed in a similar fashion between psychiatric populations and other non-psychiatric groups [71], which prompts the relevance of our method to create a gender score, and the need to replicate it in other samples.

In addition, our sample is slightly skewed regarding sex representation with 39.4% of birth-assigned females. This could influence the process to create our gender score by offering more “weight” to masculine indicators, despite balanced male/female centered variables in our final model. Furthermore, this could also explain the overlap in our gender scores between males and females as presented in Fig 3. As the score is built using mostly males’ data, the overall score is slightly skewed towards masculinity. This skewed ratio is also probably due to the over-representation of psychotic disorders in our population with a 2.3:1 ratio which is higher than what is reported in the literature, but not uncommon [28]. Furthermore, this 60:40 male–female ratio is usually balanced enough to allow statistical inferences without affecting too much statistical power, especially considering our sample size [96].

The clinical relevance of differences observed is limited to our unique sample. As presented in several studies, statistically significant results are not always reflected clearly in a clinical setting, limiting the scope of obtained results. In our case, the measures used are not always tools used for diagnostic purposes, especially our impulsivity measures. However, some scales available could be used as diagnostic tools or screeners, such as the PHQ-9 or the STAI-6. We can observe that differences between our masculine/male group (PHQ-9 score = 10), and feminine/female group (PHQ-9 score = 15) reflects a severity qualified as “mild to moderate” depression (mild = 5–9, moderate = 10–14) for masculine males, and “moderately severe” (moderately severe = 15–19) for feminine females [50].

Regarding anxious symptoms, using the norms developed by Gauthier & Bouchard [47], we can underline the fact that all our groups are over the 75^th^ percentile on the scale, indicating clinical levels of state anxiety. Our masculine/male and undifferentiated/male groups are at the 83^rd^ percentile which is significantly lower than undifferentiated females (92^nd^ percentile), feminine males (93^rd^ percentile), and feminine females (94^th^ percentile). This 11-percentile difference between our lowest and highest groups could be considered a clinically significant difference depending on both gender and BAS.

### Future directions

Our results demonstrate that it is possible to create a score reflecting sociocultural gender dimensions in an already available dataset. It will be necessary to reproduce our method using other databases to confirm the validity and utility of this method. Furthermore, these future projects will need to address the limitations of our project, being the sex distribution of the sample, as well as using a non-psychiatric sample to confirm its generalizability and relevance. This composite gender score would also greatly benefit from comparisons with validated measures of gender, but also from its use among gender diverse individuals to offer a cross-validation. Finally, research in mental health, and especially psychiatry, tends to focus on biological differences and not acknowledge the social determinants embedded in psychopathology [97]. Gender is a major social determinant of health [98], and measuring it represents an essential avenue to better understand health as a whole and inseparable dimension of the human experience.

## Conclusion

Using a data-based approach, we created a composite gender score that partially overlaps with BAS. This gender score allowed for the identification of meaningful differences in psychiatric symptom severity that were not identified when looking solely at sex. Diagnostic repartition was also different whether looking at sex or gender. Considering both sex and gender could offer a deeper understanding of the lived experiences of psychiatric patients. As a result, we replicated a method to measure gender in research, even in ongoing projects. While applied here to measure gender in psychiatric research, this method has limitations that need to be considered and refined in future research to confirm this method’s validity, replicability, and generalizability. In summary, evaluating gender could allow for the development of more personalized and tailored psychotherapy, leading to better care for psychiatric patients but also for the general population.

## References

[1] van Anders SM. Gender/sex/ual diversity and biobehavioral research. Psychol Sex Orientat Gend Divers. 2022; No Pagination Specified-No Pagination Specified. doi: 10.1037/sgd0000609

[2] Hyde JS, Bigler RS, Joel D, Tate CC, van Anders SM. The future of sex and gender in psychology: Five challenges to the gender binary. Am Psychol. 2019;74: 171–193. doi: 10.1037/amp0000307 30024214

[3] CIHR—IGH. What is gender? What is sex? In: Canadian Institutes of Health Research [Internet]. 2023 [cited 26 Jul 2023]. Available: https://cihr-irsc.gc.ca/e/48642.html.

[4] Darmstadt GL, Heise L, Gupta GR, Henry S, Cislaghi B, Greene ME, et al. Why now for a Series on gender equality, norms, and health? Lancet. 2019;393: 2374–2377. doi: 10.1016/S0140-6736(19)30985-7 31155268

[5] Lundberg U. Stress hormones in health and illness: The roles of work and gender. Psychoneuroendocrinology. 2005;30: 1017–1021. doi: 10.1016/j.psyneuen.2005.03.014 15963652

[6] Gurram S, Ashley R. Girls to boys to older men, the interesting tale of the Guevedoces and 5-alpha reductase inhibitors. J Urol. 2016;195. doi: 10.1016/j.juro.2016.02.087

[7] Blackless M, Charuvastra A, Derryck A, Fausto-Sterling A, Lauzanne K, Lee E. How sexually dimorphic are we? Review and synthesis. Am J Hum Biol. 2000;12: 151–166. doi: 10.1002/(SICI)1520-6300(200003/04)12:2&lt;151::AID-AJHB1&gt;3.0.CO;2-F 11534012

[8] National Academies of Sciences Engineering and Medicine. Measuring Sex, Gender Identity, and Sexual Orientation. Beckerz T, editor. Measuring Sex, Gender Identity, and Sexual Orientation. Washington, D.C.: National Academies Press; 2022. doi: 10.17226/26424 35286054

[9] Juster R-P, Raymond C, Desrochers AB, Bourdon O, Durand N, Wan N, et al. Sex hormones adjust “sex-specific” reactive and diurnal cortisol profiles. Psychoneuroendocrinology. 2016;63: 282–290. doi: 10.1016/j.psyneuen.2015.10.012 26539966

[10] Junker A, Juster R-P, Picard M. Integrating sex and gender in mitochondrial science. Curr Opin Physiol. 2022;26: 100536. doi: 10.1016/j.cophys.2022.100536

[11] Bem SL. The measurement of psychological androgyny. J Consult Clin Psychol. 1974;42: 155–162. doi: 10.1037/h0036215 4823550

[12] Cook EP. Psychological androgyny. 1985.

[13] Heilbrun AB. Measurement of masculine and feminine sex role identities as independent dimensions. J Consult Clin Psychol. 1976;44: 183–190. doi: 10.1037/0022-006X.44.2.183

[14] Berger A, Krahé B. Negative attributes are gendered too: Conceptualizing and measuring positive and negative facets of sex‐role identity. Eur J Soc Psychol. 2013;43: 516–531. doi: 10.1002/ejsp.1970

[15] Wester SR, Vogel DL, Pressly PK, Heesacker M. Sex Differences in Emotion. Couns Psychol. 2002;30: 630–652. doi: 10.1177/00100002030004008

[16] Curci A, Rimé B. The temporal evolution of social sharing of emotions and its consequences on emotional recovery: A longitudinal study. Emotion. 2012;12. doi: 10.1037/a0028651 22642349

[17] Johnson JL, Greaves L, Repta R. Better science with sex and gender: Facilitating the use of a sex and gender-based analysis in health research. Int J Equity Health. 2009;8: 14. doi: 10.1186/1475-9276-8-14 19419579 PMC2689237

[18] Pilote L, Raparelli V, Noris CM. Meet the Methods Series: Methods for Prospectively and Retrospectively Incorporating Gender-related Variables in Clinical Research. Can Institutes Heal Res Inst Gend Heal. 2021; 1–3. Available: https://cihr-irsc.gc.ca/e/52608.html. Accessed 25/10/2021.

[19] Howard LM, Ehrlich AM, Gamlen F, Oram S. Gender-neutral mental health research is sex and gender biased. The Lancet Psychiatry. 2017;4: 9–11. doi: 10.1016/S2215-0366(16)30209-7 27856394

[20] Afifi M. Gender differences in mental health. Singapore Med J. 2007;48: 385–391. Available: http://smj.sma.org.sg/4805/4805ra1.pdf%5Cnhttp://ovidsp.ovid.com/ovidweb.cgi?T=JS&PAGE=reference&D=emed8&NEWS=N&AN=2008077873. 17453094

[21] Kidd SA, Howison M, Pilling M, Ross LE, McKenzie K. Severe Mental Illness in LGBT Populations: A Scoping Review. Psychiatr Serv. 2016;67: 779–783. doi: 10.1176/appi.ps.201500209 26927576 PMC4936529

[22] Einstein G, Downar J, Kennedy S. Gender/sex differences in emotions. Medicographia. 2013;35: 271–280.

[23] Regitz-Zagrosek V, Kararigas G. Mechanistic pathways of sex differences in cardiovascular disease. Physiol Rev. 2017;97: 1–37. doi: 10.1152/physrev.00021.2015 27807199

[24] Diflorio A, Jones I. Is sex important? Gender differences in bipolar disorder. Int Rev Psychiatry. 2010;22: 437–452. doi: 10.3109/09540261.2010.514601 21047158

[25] Schwartz DH, Romans SE, Meiyappan S, De Souza MJ, Einstein G. The role of ovarian steroid hormones in mood. Horm Behav. 2012;62: 448–454. doi: 10.1016/j.yhbeh.2012.08.001 22902271

[26] Busfield J. Gender and mental health. The Palgrave Handbook of Gender and Healthcare, Second Edition. 2012. pp. 192–208. doi: 10.1057/9781137295408_12

[27] Duchesne A, Pletzer B, Pavlova MA, Lai M-C, Einstein G. Editorial: Bridging Gaps Between Sex and Gender in Neurosciences. Front Neurosci. 2020;14: 1–4. doi: 10.3389/fnins.2020.00561 32595445 PMC7301887

[28] Mendrek A, Mancini-Marïe A. Sex/gender differences in the brain and cognition in schizophrenia. Neurosci Biobehav Rev. 2016;67: 57–78. doi: 10.1016/j.neubiorev.2015.10.013 26743859

[29] Goldblum ED. The Oxford Handbook of Sexual and Gender Minority Mental Health. Oxford University Press. New York; 2020.

[30] Rydberg Sterner T, Gudmundsson P, Falk H, Seidu N, Ahlner F, Wetterberg H, et al. Depression in relation to sex and gender expression among Swedish septuagenarians—Results from the H70 study. Schmaling KB, editor. PLoS One. 2020;15: e0238701. doi: 10.1371/journal.pone.0238701 32925927 PMC7489509

[31] Hartung CM, Lefler EK. Sex and gender in psychopathology: DSM-5 and beyond. Psychol Bull. 2019;145: 390–409. doi: 10.1037/bul0000183 30640497

[32] Vafaei A, Ahmed T, Freire A do NF, Zunzunegui MV, Guerra RO. Depression, Sex and Gender Roles in Older Adult Populations: The International Mobility in Aging Study (IMIAS). Ginsberg SD, editor. PLoS One. 2016;11: e0146867. doi: 10.1371/journal.pone.0146867 26771828 PMC4714885

[33] Lefler EK, Tabler J, Abu-Ramadan TM, Stevens AE, Serrano JW, Shelton CR, et al. Sex, Gender, and Sexual Orientation in Psychological Research: Exploring Data Trends & Researcher Opinions. Psychol Rep. 2023;0: 1–28. doi: 10.1177/00332941231199959 37670683

[34] Arcand M, Bilodeau-Houle A, Juster R-P, Marin M-F. Sex and gender role differences on stress, depression, and anxiety symptoms in response to the COVID-19 pandemic over time. Front Psychol. 2023;14. doi: 10.3389/fpsyg.2023.1166154 37207028 PMC10189052

[35] Rossi M. Les traits de personnalité modèrent la relation entre les rôles de genre et les symptômes psychiatriques. Université de Montréal. 2022.

[36] Pelletier R, Ditto B, Pilote L. A Composite Measure of Gender and Its Association With Risk Factors in Patients With Premature Acute Coronary Syndrome. Psychosom Med. 2015;77: 517–526. doi: 10.1097/PSY.0000000000000186 25984818

[37] Smith PM, Koehoorn M. Measuring gender when you don’t have a gender measure: constructing a gender index using survey data. Int J Equity Health. 2016;15: 82. doi: 10.1186/s12939-016-0370-4 27233478 PMC4884354

[38] Lupien SJ, Sasseville M, François N, Giguère CE, Boissonneault J, Plusquellec P, et al. The DSM5/RDoC debate on the future of mental health research: implication for studies on human stress and presentation of the signature bank. Stress. 2017;20: 95–111. doi: 10.1080/10253890.2017.1286324 28124571

[39] Kerr P, Le Page C, Giguère C-É, Marin M-F, Trudel-Fitzgerald C, Romain AJ, et al. The Signature Biobank: A Longitudinal Biopsychosocial Repository of Psychiatric Emergency Patients. Psychiatry Res. doi: 10.1016/j.psychres.2024.115718 38198857

[40] Gravel R, Béland Y. The Canadian Community Health Survey: Mental Health and Well-Being. Can J Psychiatry. 2005;50: 573–579. doi: 10.1177/070674370505001002 16276847

[41] Limoges É, Mottron L, Bolduc C, Berthiaume C, Godbout R. Atypical sleep architecture and the autism phenotype. Brain. 2005;128: 1049–1061. doi: 10.1093/brain/awh425 15705609

[42] Poulin J, Chouinard S, Pampoulova T, Lecomte Y, Stip E, Godbout R. Sleep habits in middle-aged, non-hospitalized men and women with schizophrenia: A comparison with healthy controls. Psychiatry Res. 2010;179: 274–278. doi: 10.1016/j.psychres.2009.08.009 20493544

[43] Tanaka M, Wekerle C, Leung E, Waechter R, Gonzalez A, Jamieson E, et al. Preliminary Evaluation of the Childhood Experiences of Violence Questionnaire Short Form. J Interpers Violence. 2012;27: 396–407. doi: 10.1177/0886260511416462 21810788

[44] Walsh CA, MacMillan HL, Trocmé N, Jamieson E, Boyle MH. Measurement of victimization in adolescence: Development and validation of the Childhood Experiences of Violence Questionnaire. Child Abuse Negl. 2008;32: 1037–1057. doi: 10.1016/j.chiabu.2008.05.003 18992940

[45] Brown GL, Goodwin FK. Cerebrospinal Fluid Correlates of Suicide Attempts and Aggression. Ann N Y Acad Sci. 1986;487: 175–188. doi: 10.1111/j.1749-6632.1986.tb27897.x 2436532

[46] Dellazizzo L, Potvin S, Giguère CÉ, Berwald M, Dugré JR, Dumais A. The psychometric properties of the Life History of Aggression evaluated in patients from a psychiatric emergency setting. Psychiatry Res. 2017;257: 485–489. doi: 10.1016/j.psychres.2017.08.031 28841510

[47] Gauthier J, Bouchard S. Adaptation Canadienne-Française de la forme révisée du State-Trait Anxiety Inventory de Spielberger. Can J Behav Sci. 1993;25: 559–578. doi: 10.1037/h0078881

[48] Marteau TM, Bekker H. The development of a six‐item short‐form of the state scale of the Spielberger State—Trait Anxiety Inventory (STAI). Br J Clin Psychol. 1992;31: 301–306. doi: 10.1111/j.2044-8260.1992.tb00997.x 1393159

[49] Spielberger CD. State-Trait Anxiety Inventory. The Corsini Encyclopedia of Psychology. Hoboken, NJ, USA: John Wiley & Sons, Inc.; 2010. doi: 10.1002/9780470479216.corpsy0943

[50] Kroenke K, Spitzer RL, Williams JBW. The PHQ-9. J Gen Intern Med. 2001;16: 606–613. doi: 10.1046/j.1525-1497.2001.016009606.x 11556941 PMC1495268

[51] Billieux J, Rochat L, Ceschi G, Carré A, Offerlin-Meyer I, Defeldre AC, et al. Validation of a short French version of the UPPS-P Impulsive Behavior Scale. Compr Psychiatry. 2012;53: 609–615. doi: 10.1016/j.comppsych.2011.09.001 22036009

[52] Dugré JR, Giguére C-É, Percie du Sert O, Potvin S, Dumais A. The Psychometric Properties of a Short UPPS-P Impulsive Behavior Scale Among Psychiatric Patients Evaluated in an Emergency Setting. Front Psychiatry. 2019;10: 1–9. doi: 10.3389/fpsyt.2019.00139 30967798 PMC6442540

[53] Whiteside SP, Lynam DR. Understanding the role of impulsivity and externalizing psychopathology in alcohol abuse: Application of the UPPS Impulsive Behavior Scale. Personal Disord Theory, Res Treat. 2009;S: 69–79. doi: 10.1037/1949-2715.s.1.69 12940500

[54] Bebbington P, Nayani T. The psychosis screening questionnaire. Int J Methods Psychiatr Res. 1995.

[55] Saunders JB, Aasland OG, Babor TF, De La Fuente JR, Grant M. Development of the Alcohol Use Disorders Identification Test (AUDIT): WHO Collaborative Project on Early Detection of Persons with Harmful Alcohol Consumption-II. Addiction. 1993;88: 791–804. doi: 10.1111/j.1360-0443.1993.tb02093.x 8329970

[56] Skinner HA. The drug abuse screening test. Addict Behav. 1982;7: 363–371. doi: 10.1016/0306-4603(82)90005-3 7183189

[57] Giguère C-É, Potvin S, The Signature Consortium. The Drug Abuse Screening Test preserves its excellent psychometric properties in psychiatric patients evaluated in an emergency setting. Addict Behav. 2017;64: 165–170. doi: 10.1016/j.addbeh.2016.08.042 27614056

[58] Üstün TB, Chatterji S, Kostanjsek N, Rehm J, Kennedy C, Epping-J. Developing the World Health Organization Disability Assessment Schedule 2. 0. 2010; 815–823. doi: 10.2471/BLT.09.067231 21076562 PMC2971503

[59] Hoehne A, Giguère C-E, Herba CM, Labelle R. Assessing Functioning across Common Mental Disorders in Psychiatric Emergency Patients: Results from the WHODAS-2. Can J Psychiatry. 2021;66: 1085–1093. doi: 10.1177/0706743720981200 33353429 PMC8689447

[60] World Health Organization (WHO). ICD-10: international statistical classification of diseases and related health problems: tenth revision. Geneva PP—Geneva: World Health Organization; 2004. Available: https://apps.who.int/iris/handle/10665/42980.

[61] Rosseel Y, Oberski D, Byrnes J, Vanbrabant L, Savalei V, Merkle E, et al. Package ‘lavaan.’ Retrieved June. 2017;17: 2017.

[62] Revelle W. Package ‘psych.’ Compr R Arch Netw. 2015;337: 338.

[63] Carothers BJ, Reis HT. Men and women are from Earth: Examining the latent structure of gender. J Pers Soc Psychol. 2013;104: 385–407. doi: 10.1037/a0030437 23088230

[64] Beck F, Legleye S, Maillochon F, de Peretti G. Femmes influentes sous influence? médecine/sciences. 2010;26: 95–97. doi: 10.1051/medsci/201026195 20132782

[65] Schoon I, Parsons S. Teenage aspirations for future careers and occupational outcomes. J Vocat Behav. 2002;60: 262–288. doi: 10.1006/jvbe.2001.1867

[66] Hooper D, Coughlan J, Mullen MR. Structural Equation Modeling: Guidelines for Determining Model Fit Structural Equation Modelling: Guidelines for Determining Model Fit. Electron J Bus Res Methods. 2008;6: 53–60. doi: 10.21427/D79B73

[67] Dziuban CD, Shirkey EC. When is a correlation matrix appropriate for factor analysis? Some decision rules. Psychol Bull. 1974;81: 358–361. doi: 10.1037/h0036316

[68] Mardia K V. Measures of multivariate skewness and kurtosis with applications. Biometrika. 1970;57: 519–530. doi: 10.1093/biomet/57.3.519

[69] Field A, Miles J, Field Z. Discovering statistics using R. Sage publications; 2012.

[70] Ferrer-pérez VA, Bosch-fiol E. The measure of the masculinity–femininity construct today: Some reflections on the case of the Bem Sex Role Inventory / La medida del constructo masculinidad–feminidad en la actualidad: algunas reflexiones sobre el caso del Bem Sex Role Inventory. Rev Psicol Soc / Int J Soc Psychol. 2014;29: 180–207. doi: 10.1080/02134748.2013.878569

[71] Evans RG, Dinning WD. MMPI correlates of the Bem Sex Role Inventory and extended personal attributes questionnaire in a male psychiatric sample. J Clin Psychol. 1982;38: 811–815. doi: 10.1002/1097-4679(198210)38:4&lt;811::aid-jclp2270380420&gt;3.0.co;2-o 7174815

[72] Loree AM, Lundahl LH, Ledgerwood DM. Impulsivity as a predictor of treatment outcome in substance use disorders: Review and synthesis. Drug Alcohol Rev. 2015;34: 119–134. doi: 10.1111/dar.12132 24684591

[73] Dawe S, Loxton NJ. The role of impulsivity in the development of substance use and eating disorders. Neurosci Biobehav Rev. 2004;28: 343–351. doi: 10.1016/j.neubiorev.2004.03.007 15225976

[74] Ramírez-Martín A, Ramos-Martín J, Mayoral-Cleries F, Moreno-Küstner B, Guzman-Parra J. Impulsivity, decision-making and risk-taking behaviour in bipolar disorder: A systematic review and meta-analysis. Psychol Med. 2020;50: 2141–2153. doi: 10.1017/S0033291720003086 32878660

[75] American Psychiatric Association. Diagnostic and Statistical Manual of Mental Disorders 5-TR. American Psychiatric Association Publishing; 2022. doi: 10.1176/appi.books.9780890425787

[76] Carvalho AF, Heilig M, Perez A, Probst C, Rehm J. Alcohol use disorders. Lancet. 2019;394: 781–792. doi: 10.1016/S0140-6736(19)31775-1 31478502

[77] Canada Statistics. Mental health indicators. 2014. doi: 10.25318/1310046501-eng

[78] Zakiniaeiz Y, Potenza MN. Gender-related differences in addiction: a review of human studies. Curr Opin Behav Sci. 2018;23: 171–175. doi: 10.1016/j.cobeha.2018.08.004 PMC11784943 39896826

[79] Pinkhasov RM, Wong J, Kashanian J, Lee M, Samadi DB, Pinkhasov MM, et al. Are men shortchanged on health? Perspective on health care utilization and health risk behavior in men and women in the United States. Int J Clin Pract. 2010;64: 475–487. doi: 10.1111/j.1742-1241.2009.02290.x 20456194

[80] Cross CP, Copping LT, Campbell A. Sex Differences in Impulsivity: A Meta-Analysis. 2011;137: 97–130. doi: 10.1037/a0021591 21219058

[81] Cyders MA. Impulsivity and the Sexes: Measurement and Structural Invariance of the UPPS-P Impulsive Behavior Scale. 2013. doi: 10.1177/1073191111428762 22096214

[82] McHugh RK, Votaw VR, Sugarman DE, Greenfield SF. Sex and gender differences in substance use disorders. Clin Psychol Rev. 2018;66: 12–23. doi: 10.1016/j.cpr.2017.10.012 29174306 PMC5945349

[83] Tibubos AN, Otten D, Ernst M, Beutel ME. A Systematic Review on Sex- and Gender-Sensitive Research in Public Mental Health During the First Wave of the COVID-19 Crisis. Front Psychiatry. 2021;12. doi: 10.3389/fpsyt.2021.712492 34603104 PMC8484908

[84] Lengua LJ, Stormshak EA. Gender, gender roles, and personality: Gender differences in the prediction of coping and psychological symptoms. Sex Roles. 2000;43: 787–820. doi: 10.1023/A:1011096604861

[85] Fattore L, Melis M. Editorial: Exploring Gender and Sex Differences in Behavioral Dyscontrol: From Drug Addiction to Impulse Control Disorders. Front Psychiatry. 2016;7: 1121–1155. doi: 10.3389/fpsyt.2016.00019 26941657 PMC4762070

[86] Remes O, Brayne C, van der Linde R, Lafortune L. A systematic review of reviews on the prevalence of anxiety disorders in adult populations. Brain Behav. 2016;6: e00497. doi: 10.1002/brb3.497 27458547 PMC4951626

[87] Bandelow B, Michaelis S. Epidemiology of anxiety disorders in the 21st century. Dialogues Clin Neurosci. 2015;17: 327–335. doi: 10.31887/DCNS.2015.17.3/bbandelow 26487813 PMC4610617

[88] Deslauriers J-M, Baron M, Negura L. Les hommes souffrant de dépression: un problème méconnu. Réalités masculines oubliées. 2019; 183–214.

[89] Arcand M, Juster R-P, Lupien SJ, Marin M-F. Gender roles in relation to symptoms of anxiety and depression among students and workers. Anxiety, Stress Coping. 2020;33: 661–674. doi: 10.1080/10615806.2020.1774560 32490683

[90] Lin J, Zou L, Lin W, Becker B, Yeung A, Cuijpers P, et al. Does gender role explain a high risk of depression? A meta-analytic review of 40 years of evidence. J Affect Disord. 2021;294: 261–278. doi: 10.1016/j.jad.2021.07.018 34304081

[91] Bangasser DA, Cuarenta A. Sex differences in anxiety and depression: circuits and mechanisms. Nat Rev Neurosci. 2021;22: 674–684. doi: 10.1038/s41583-021-00513-0 34545241

[92] Tyrer P, Reed GM, Crawford MJ. Classification, assessment, prevalence, and effect of personality disorder. Lancet. 2015;385: 717–726. doi: 10.1016/S0140-6736(14)61995-4 25706217

[93] Bjorklund P. No man’s land: gender bias and social constructivismin the diagnosis of borderline personality disorder. Issues Ment Health Nurs. 2006;27: 3–23. doi: 10.1080/01612840500312753 16352513

[94] Horsfall J. Gender and mental illness: An australian overview. Issues Ment Health Nurs. 2001;22: 421–438. doi: 10.1080/01612840119734 11885157

[95] Nielsen MW, Stefanick ML, Peragine D, Neilands TB, Ioannidis JPA, Pilote L, et al. Gender-related variables for health research. Biol Sex Differ. 2021;12: 23. doi: 10.1186/s13293-021-00366-3 33618769 PMC7898259

[96] Faul F, Erdfelder E, Lang A-G, Buchner A. G*Power 3: A flexible statistical power analysis program for the social, behavioral, and biomedical sciences. Behav Res Methods. 2007;39: 175–191. doi: 10.3758/bf03193146 17695343

[97] Di Nicola V. “A person is a person through other persons”: A social psychiatry manifesto for the 21 st century. World Soc Psychiatry. 2019;1: 8. doi: 10.4103/WSP.WSP_11_19

[98] Raphael D, Bryant T, Mikkonen J, A. R. Social Determinants of Health: The Canadian Facts. 2nd ed. York University School of Health Policy and Management. Oshawa: Ontario Tech University Faculty of Health Sciences; 2020. Available: http://www.thecanadianfacts.org/.

